# Patient-Derived Anti-NMDAR Antibody Disinhibits Cortical Neuronal Networks through Dysfunction of Inhibitory Neuron Output

**DOI:** 10.1523/JNEUROSCI.1689-21.2022

**Published:** 2022-04-13

**Authors:** Ewa Andrzejak, Eshed Rabinovitch, Jakob Kreye, Harald Prüss, Christian Rosenmund, Noam E. Ziv, Craig C. Garner, Frauke Ackermann

**Affiliations:** ^1^German Center for Neurodegenerative Diseases, Berlin 10117, Germany; ^2^Technion Faculty of Medicine, Rappaport Institute and Network Biology Research Laboratories, Fishbach Building, Technion City, Haifa 32000, Israel; ^3^Department of Neurology and Experimental Neurology, Charité – Universitätsmedizin, Berlin 10117, Germany; ^4^Institute of Neurophysiology, Charité – Universitätsmedizin, Berlin 10117, Germany; ^5^NeuroCure Cluster of Excellence, Charité – Universitätsmedizin, Berlin 10117, Germany

**Keywords:** autoantibodies, autoimmune encephalitis, cortical interneurons, network excitability, NMDAR

## Abstract

Anti-NMDA receptor (NMDAR) encephalitis is a severe neuropsychiatric disorder associated with autoantibodies against NMDARs, which cause a variety of symptoms from prominent psychiatric and cognitive manifestations to seizures and autonomic instability. Previous studies mainly focused on hippocampal effects of these autoantibodies, helping to explain mechanistic causes for cognitive impairment. However, antibodies' effects on higher cortical network function, where they could contribute to psychosis and/or seizures, have not been explored in detail until now. Here, we employed a patient-derived monoclonal antibody targeting the NR1 subunit of NMDAR and tested its effects on *in vitro* cultures of rodent cortical neurons, using imaging and electrophysiological techniques. We report that this hNR1 antibody drives cortical networks to a hyperexcitable state and disrupts mechanisms stabilizing network activity such as Npas4 signaling. Network hyperactivity is in part a result of a reduced synaptic output of inhibitory neurons, as indicated by a decreased inhibitory drive and levels of presynaptic inhibitory proteins, specifically in inhibitory-to-excitatory neuron synapses. Importantly, on a single-cell level hNR1 antibody selectively impairs NMDAR-mediated currents and synaptic transmission of cortical inhibitory neurons, yet has no effect on excitatory neurons, which contrasts with its effects on hippocampal neurons. Together, these findings provide a novel, cortex-specific mechanism of antibody-induced neuronal hyperexcitability, highlighting regional specificity underlying the pathology of autoimmune encephalitis.

**SIGNIFICANCE STATEMENT** It is increasingly appreciated that the inadvertent activation of the immune system within CNS can underlie pathogenesis of neuropsychiatric disorders. Although the exact mechanisms remain elusive, autoantibodies derived from patients with autoimmune encephalitis pose a unique tool to study pathogenesis of neuropsychiatric states. Our analysis reveals that autoantibody against the NMDA receptor (NMDAR) has a distinct mechanism of action in the cortex, where it impairs function of inhibitory neurons leading to increased cortical network excitability, in contrast to previously described hippocampal synaptic mechanisms of information encoding, highlighting brain regional specificity. Notably, similar mechanism of NMDAR-mediated inhibitory hypofunction leading to cortical disinhibition has been suggested to underlie pathology of schizophrenia, hence our data provide new evidence for common mechanisms underlying neuropsychiatric disorders.

## Introduction

Over the last decade, a growing number of central nervous system disorders have been linked to autoantibodies, which have been detected in patients' cerebrospinal fluid (CSF). Many of these bind synaptic and neuronal cell-surface proteins, including a variety of neuro-transmitter receptors (Dalmau and Graus, 2018). One of the most prevalent forms of autoimmune encephalitis is associated with IgG antibodies that bind NMDA receptors (NMDARs), a disease that is characterized by a rapid clinical progression and a broad range of symptoms (Dalmau et al., 2007). Most patients present with prominent psychiatric manifestations, including psychosis, hallucinations, and behavioral changes, which further progress to severe memory loss, seizures, and autonomic instability. Often patients require prolonged treatment in intensive care units (Irani et al., 2010). Interestingly, most are responsive to immunotherapies and show marked recovery (Titulaer et al., 2013). A fundamental question is how these autoantibodies mechanistically trigger such a broad range of symptoms in patients.

With regard to NMDAR encephalitis, most studies have focused on how autoantibodies cause memory deficits, in particular within hippocampal circuits. A framework for such investigations is based on a long-history of research showing that NMDARs are ligand-gated ion channels responsible for synaptic integration and plasticity and underlie hippocampal learning and memory processes (Bliss and Collingridge, 1993; Nicoll, 2017). Studies with patients' CSF reveal that most NMDAR autoantibodies recognize epitopes within the extracellular domain of the obligatory NR1 subunit of NMDARs (Gleichman et al., 2012). Antibody binding primarily triggers the reversible internalization of NMDARs and thereby a net decrease in surface receptor clusters in hippocampal neurons (Hughes et al., 2010; Planagumà et al., 2015). Loss of receptors has been shown to decrease synaptic NMDAR-mediated currents (Hughes et al., 2010; Kreye et al., 2016) and to compromise synaptic plasticity (Mikasova et al., 2012; Zhang et al., 2012; Würdemann et al., 2016). These conditions are thought to contribute to memory deficits described in patients with NMDAR encephalitis and corresponding animal models (Planagumà et al., 2016; Malviya et al., 2017).

At present, how such antibodies could also trigger psychiatric symptoms and/or epileptic seizures is less clear, although actions at higher network levels, for instance within cortical circuits, seem likely. Intriguingly, seizures have been observed in ∼60% of encephalitis patients (de Bruijn et al., 2019), suggesting an increased excitability within these circuits (Manto et al., 2010). Yet there are few clues of how NMDARs autoantibodies could drive this cortical hyperexcitability. On the other hand, it has long been suggested that NMDARs are involved in the pathology of psychosis (“glutamatergic hypothesis of schizophrenia”), wherein NMDAR antagonists such as ketamine and phencyclidine (PCP) have been found to induce psychotic symptoms, as well as exacerbate them in schizophrenic patients (Krystal et al., 1994; Jentsch and Roth, 1999). This has led to the hypothesis that NMDAR hypofunction, in particular on inhibitory interneurons (Belforte et al., 2010), could also cause excitation/inhibition imbalances and disinhibition of prefrontal cortical circuits (Homayoun and Moghaddam, 2007), similar to those observed in schizophrenic patients (Uhlhaas and Singer, 2010). It is currently unclear, whether anti-NMDAR auto antibodies could similarly affect cortical interneurons and thus alter cortical network properties in patients with NMDAR encephalitis.

In the present study, we have evaluated whether a patient-derived antibody against the NR1 subunit of NMDARs has direct effects on cortical neurons. Our detailed imaging and electrophysiological analysis of cultured rodent cortical neurons revealed that this antibody increases network spiking and bursting, driving cortical neurons into a hyperactive state. Surprisingly, this hyperactivity seems to be a result of selective hypofunction of inhibitory interneuron output, specifically in inhibitory-to-excitatory neuron synapses, leading to a decrease in inhibitory drive and hyperexcitability of these cortical neuronal networks.

## Materials and Methods

### Preparation of cultured cortical neurons

All procedures for experiments involving animals were approved by the animal welfare committee of Charité Medical University and the Berlin state government. To distinguish inhibitory and excitatory neurons in some experiments, we used GAD67-GFP (Δneo)/+ (GAD67-GFP) mice, in which inhibitory neurons expressing GAD67 are fluorescently labeled (Tamamaki et al., 2003). In comparison to WT neurons, no changes in the electrophysiological properties of these GFP-expressing neurons have been reported (Chang et al., 2014). To prepare primary cortical neuronal cultures for immunocytochemistry and mass culture patch-clamp experiments, mouse WT (C57BL/6J; RRID:IMSR_JAX:000664; license: T0036/14) or GAD67-GFP animals (license: T0220/09) of either sex were prepared and grown on glass coverslips using the Banker Protocol (Banker and Goslin, 1988; Meberg and Miller, 2003). In brief, astrocytes were prepared from mouse WT cortices on postnatal day (P)0–P2 and seeded on 6-well or 12-well plates at a density of 10,000/1 cm^2^, 7–11 d before the addition of neurons. Cortical neurons were prepared from cortices dissected from mice P0–P2 brains in cold HBSS (Millipore), followed by a 45-min incubation in enzyme solution containing DMEM (Invitrogen, Thermo Fisher Scientific), cysteine (3.3 mm), CaCl_2_ (2 mm), EDTA (1 mm), and papain (20 U/ml, Worthington) at 37°C. Next, the papain reaction was inhibited by incubating cortical tissue in a DMEM solution containing 10% fetal calf serum (Thermo Fisher Scientific), bovine serum albumin (38 mm, Sigma-Aldrich), and trypsin inhibitor (95 mm; Sigma-Aldrich) for 5 min. Afterwards, cells were triturated in Neurobasal-A medium (2% B-27, 1% Glutamax, 0.2% P/S; Thermo Fisher Scientific) by gentle pipetting up and down. Isolated neuronal cells were plated onto nitric acid washed and poly-L-lysine-coated glass coverslips with paraffin dots, at a density of 50,000–200,000 per 12-well coverslip. After 1.5 h, the coverslips were placed upside down onto a bed of astrocytes and co-cultured in Neurobasal-A medium at 37°C, 5% CO_2_, for 14–18 days *in vitro* (DIV) before starting experiments.

To create autaptic cultures for single-cell electrophysiological recordings, cortical or hippocampal neurons were plated on microislands of astrocyte feeder layers, generated one week before the neuronal culture preparations, as described previously (Arancillo et al., 2013). Neurons were plated at a lower density (4000 cells per six-well coverslip) to obtain a single neuron on an astrocytic island.

For calcium imaging experiments, primary mouse cortical neurons were plated on top of a one-week-old astrocyte feeder layer, on round dishes containing a cell location grid at the bottom (µ-Dish 35 mm, high Grid-500, Ibidi), at the density of 250,000 cells per dish. The cells were cultured in medium containing minimal essential medium (MEM; Sigma-Aldrich), insulin (25 mg/l, Sigma-Aldrich), glucose (20 mm, Sigma-Aldrich), l-glutamine (2 mm, Sigma-Aldrich), Pen/Strep (0.2%, Sigma-Aldrich), and NuSerum (10%, Becton Dickinson Labware). Seven days after plating, one-half of the culture medium was replaced with feeding medium as above, but lacking NuSerum and containing 0.5 mm l-glutamine and 2% B-27 supplement (Invitrogen). Approximately one-half of the medium was then replaced with the feeding medium one or two times per week.

For multielectrode array (MEA) experiments, primary cultures of rat cortical neurons were prepared as described previously (Minerbi et al., 2009), using a protocol approved by the Technion committee for the supervision of animal experiments (IL-116-08-71). Briefly, cortices of newborn P1 Wistar rats (either sex; Charles River Laboratories) were dissected, dissociated by trypsin treatment followed by trituration using a siliconized Pasteur pipette. A total of 1–1.5 × 10^6^ cells were then plated on thin-glass MEA dishes (Multi Channel Systems, MCS), precoated with polyethylenimine (Sigma-Aldrich) for better cell adhesion. Subsequently, neurons were cultured at 37°C and 5% CO_2_, and grown in medium containing MEM (Sigma-Aldrich), insulin (25 mg/l, Sigma-Aldrich), glucose (20 mm, Sigma-Aldrich), l-glutamine (2 mm, Sigma-Aldrich), gentamycin sulfate (5 mg/ml, Sigma-Aldrich), and 10% NuSerum (Becton Dickinson Labware). At 7 DIV, one-half of the culture medium was replaced with feeding medium lacking NuSerum and containing 0.5 mm l-glutamine and 2% B-27 supplement (Invitrogen), and then the cultures were fed in this way three to four times per week.

### Lentivirus production

All lentiviral particles were provided by the Viral Core Facility of the Charite – Universitätsmedizin, Berlin (https://vcf.charite.de/en/) and were prepared as described previously (Lois et al., 2002). Briefly, HEK293T (RRID:CVCL_0063) cells were cotransfected with 10 µg of shuttle vector, 5 µg of helper plasmid pCMVdR8.9, and 5 µg of pVSV.G with X-tremeGENE 9 DNA transfection reagent (Roche Diagnostics). Virus-containing cell culture supernatant was collected after 72 h and filtered for purification. Aliquots were flash-frozen in liquid nitrogen and stored at −80°C. Neurons were infected with lentivirus at 2–4 DIV.

### Production of monoclonal antibodies

The human monoclonal antibody #003-102 is reactive to the NR1 subunit of NMDARs and was isolated from CSF of a patient with acute NMDAR encephalitis (Kreye et al., 2016). Isotype-matched control antibody mGO53 was isolated from blood of a healthy donor (Wardemann et al., 2003). For the recombinant expression of these antibodies, the paired expression vectors encoding for the antibodies' heavy and light chain were transiently transfected in HEK293T cells and purified from cell culture supernatants, as previously described (Kreye et al., 2016). The antibody concentration was determined using an anti-human IgG ELISA following the manufacturer's instructions (3850-1AD-6, Mabtech).

### Immunocytochemistry of cultured neurons

#### Npas4 experiment

To verify the effects of the hNR1 antibody on somatic Npas4 expression, cortical neurons 14–17 DIV were incubated with 1 µg/ml of hNR1 antibody for 2, 4, or 24 h. Alternatively, to induce expression of Npas4, neurons were treated with NMDA (2 μm, Tocris) for 2 h. Untreated cells were used as a control. To study the effect of hNR1 and NMDAR antagonist (2R)-amino-5-phosphonovaleric acid (AP5) on NMDA-induced Npas4 expression, NMDA treatment was preceded by 6 h or 24 h of hNR1 (1 µg/ml) or 3 h of AP5 (100 μm, Tocris) treatment. After treatment, cells were fixed with 4% paraformaldehyde (PFA) in PBS for 10 min and washed 3 × 5 min with PBS. Subsequently, cells were permeabilized and blocked with a solution containing 5% normal goat serum, 2% BSA, and 0.1% Triton in PBS, for 1 h. Neurons were then incubated with primary antibodies against Npas4 (1:1500, rabbit, Activity Signaling) and MAP2 (1:2000, chicken, Millipore, AB5543) in antibody solution containing 2% BSA in PBS for 2 h at room temperature (RT). Cells were then washed 3 × 5 min with PBS and incubated with the differently labeled secondary antibodies (1:1000 in antibody solution, Invitrogen, Thermo Fisher Scientific), for 2 h at RT and washed 3 × 5 min with PBS. Finally, coverslips were dipped in H_2_O and mounted in Pro-Long Diamon Antifade Mountant (Thermo Fisher Scientific).

#### Synaptic/extrasynaptic hNR1 labeling

Cortical neurons of GAD67-GFP animals were subjected to live staining with hNR1 and anti-human secondary antibody. Briefly, neurons were incubated with 1 µg/ml of hNR1 in original culture NBA medium (see above) for 20 min at 10°C. Cells were then washed once with same medium and incubated with Alexa Fluor 568-conjugated anti-human secondary antibody (1:1000, Thermo Fisher Scientific) for 20 min at 10°C. Afterwards, neurons were again washed once with culture medium, fixed with 4% PFA, blocked, stained with antibodies against excitatory presynaptic vesicle protein VGLUT1 (1:4000, SySy 135304, Synaptic Systems) or the inhibitory postsynaptic scaffold protein gephyrin (1:500, SySy 147011, Synaptic Systems) and labeled with secondary antibodies and mounted as described earlier (see Npas4 experiment).

#### Inhibitory presynaptic proteins labeling

Cortical neurons from GAD67-GFP animals were incubated with 1 µg/ml of hNR1 antibody in original culture NBA medium for 0, 6, or 24 h at 37°C. Subsequently, cells were fixed with 4% PFA, blocked, stained with antibodies against MAP2 (1:2000, AB5543, Millipore), glutamate decarboxylase 65 (GAD65; 1:750, MAB5406, Millipore), or vesicular GABA transporter (VGAT; 1:4000, SySy 131003, Synaptic Systems) and labeled with secondary antibodies before mounting as described above.

### Image acquisition and quantification

Immunocytochemical staining for Npas4 experiments was acquired on a Nikon Spinning Disk Confocal CSU-X microscope equipped with an air 20× Plan Apo objective lens (NA = 0.8), controlled via NIS-Elements software (Nikon). Images were then processed using ImageJ (RRID: SCR_003070). ROIs were manually drawn around cell somas. After background subtraction, the mean intensity values of the ROIs were measured and normalized to the untreated control.

hNR1 labeling and staining for inhibitory presynaptic markers were analyzed using a spinning disk confocal microscope (Carl Zeiss Axio Oberserver.Z1 with Andor spinning disk and cobalt, omricron, i-beam laser; Carl Zeiss, Andor) using a 63× (1.4 NA) or 100× (1.4 NA) Plan-Apochromat oil objectives and an iXon ultra (Andor) camera controlled by iQ software (RRID: SCR_014461; Andor). Only dendrites in proximity to the soma were imaged, and inhibitory and excitatory dendrites were distinguished based on GFP signal. Images were processed using ImageJ (RRID: SCR_003070) and OpenView software (written by Prof. Dr. Noam Ziv, Technion Institute, Haifa, Israel). In brief, for synaptic protein levels at GAD65 and VGAT puncta along dendrites were detected with set parameters: 6 × 6-pixel boxes were placed over puncta and the mean fluorescence intensities were measured, followed by subtraction of background values. For hNR1 labeling within excitatory synapses, hNR1-positive puncta were detected in a similar manner and then VGLUT1 intensities within hNR1-positive puncta were measured. A value above a threshold of 5× background intensity was considered VGLUT1-positive and thus synaptic. The remaining hNR1 puncta were considered extrasynaptic. In a similar manner, boxes around detected gephyrin puncta served to measure hNR1 intensity for localization of hNR1 puncta to inhibitory synapses. Z-plane images were analyzed using Volume Viewer plugin in ImageJ. Values of all puncta per region of interest (individual dendrites) were averaged and considered one data point.

### MEAs

Neuronal network activity was recorded continuously from MEA electrodes (59 electrodes, 30 μm in diameter, arranged in an 8 × 8 array), as described previously (Hazan and Ziv, 2020). Briefly, cells grown on MEA dishes were continuously perfused with fresh feeding medium at a rate of 4 ml/d using an ultra-low-flow peristaltic pump (Instech Laboratories), and maintained at 37°C and 5% CO_2_. After setting up the perfusion system, network activity was allowed to equilibrate for 1 h and then 15 h of baseline activity was recorded. Next, 1 µg/ml of hNR1 antibody, control antibody or 1 μm AP5 was manually pipetted into the MEA dish and added to the perfusion system. Cell spiking was recorded in the presence of the antibodies or 1 μm AP5 for 24 h. Finally, saturating concentrations of AP5 (50 μm) were added to the dish to block remaining NMDARs, and the activity of the culture was recorded for additional 6 h. Recordings from MEA dishes were performed using a commercial 60-channel headstage (inverted MEA-1060-BC, MCS) with a gain of 53× and frequency limits of 0.02–8500 Hz. This signal was further filtered with frequency limits of 150–3000 Hz and amplified (20×) using a filter/amplifier (FA60S-BC, MCS). Data acquisition was performed using custom software [Closed Loop Experiment Manager (CLEM); Hazan and Ziv, 2017]. Data were collected at 16,000 samples per second. Action potentials were identified as negative threshold-crossing events, with the threshold calculated as 5× root-mean-square of traces recorded at the beginning of each experiment. Data were imported, converted and analyzed for spiking and bursting activity using custom scripts in MATLAB (MathWorks). Data from each MEA dish was normalized to the activity of last 3 h of baseline recordings. To test whether there is a significant increase in spiking/bursting rate because of 24-h treatment, for each network the average rate at the end of baseline (1 h) was compared with end of 24-h treatment (1 h) by paired *t* test. To quantify percentage increase in spiking/bursting rates at the beginning/end of respective treatments (24-h treatment with antibodies or 1 μm AP5, 50 μm AP5 or recovery at the end of experiment), the spike/burst rate of 10-min intervals was measured.

### Patch-clamp electrophysiology

Whole-cell patch-clamp recordings were performed on mass or autaptic, cortical or hippocampal neurons at 14–18 DIV, after 24-h incubation with 1 µg/ml of hNR1 or control antibody (mGo53; Wardemann et al., 2003). Cortical neurons from GAD67-eGFP mouse line were used to distinguish inhibitory (GFP-positive) and excitatory (GFP-negative) cells. All recordings were obtained at ∼25°C from neurons clamped at −70 mV with a Multiclamp 700B amplifier (Molecular Devices) under the control of Clampex 10.4 software (Molecular Devices). Data were sampled at 10 kHz and low-pass Bessel filtered at 3 kHz and series resistance was typically under 15 MΩ and compensated at 70%. During all recordings, except for chemically induced NMDA and evoked synaptic NMDA responses, neurons were immersed in standard extracellular solution consisting of 140 mm NaCl, 2.4 mm KCl, 10 mm HEPES, 10 mm glucose, 2 mm CaCl_2_, and 4 mm MgCl_2_. Chemically induced whole-cell NMDA responses were measured in extracellular solution containing 0 mm Mg^2+^, 0.2 mm CaCl_2_, and 10 μm glycine, whereas synaptically evoked NMDAR currents were measured in extracellular solution containing 0 mm Mg^2+^, 2 mm CaCl_2_, and 10 μm glycine. The borosilicate glass pipettes (3–8 MΩ) were pulled with a micropipette puller device (Sutter Instruments) and filled with the internal solution containing 136 mm KCl, 17.8 mm HEPES, 1 mm EGTA, 0.6 mm MgCl_2_, 4 mm ATP-Mg, 0.3 mm GTP-Na, 12 mm phosphocreatine, and 50 U/ml phosphocreatine kinase. All solutions were adjusted to pH 7.4 and osmolarity of ∼300 mOsm.

To selectively induce NMDA, GABA, and kainate currents, NMDA (10 μm, Tocris), GABA (5 μm, Tocris), or kainic acid (20 μm, Tocris), were acutely applied to the neurons for 1 s by use of a fast-flow system. For synaptic responses in autaptic cultures, EPSCs were evoked by brief somatic depolarization of neurons from −70 to 0 mV for 2 ms. Synaptic NMDAR currents were measured in the presence of AMPAR antagonist NBQX (10 μm, Tocris).

All mass culture recordings were performed in the presence of TTX (0.5 μm, Tocris) to block propagation of action potentials. To identify spontaneous miniature (m)EPSCs and mIPSCs, neurons were additionally immersed in AP5 (50 μm) and bicuculline (20 μm, Tocris), or AP5 (50 μm) and NBQX (10 μm), respectively. Traces were recorded at a holding potential of −70 mV and were filtered at 1 kHz, mEPSCs and mIPSCs were detected by a template algorithm in Axograph X (Axograph Scientific). False-positive events were excluded by subtracting events detected from traces in the presence of NBQX (10 μm) or bicuculline (20 μm) for mEPSCs and mIPSCs, respectively. To identify excitatory neurons for detection of mIPSCs in mass cultures, WT mouse neurons were infected with pLenti_CamKIIa_mKate2, a lentivirus expressing mKate2 under a CamKII promoter, at 3–4 DIV. For experiments detecting mEPSCs, GAD67-GFP line neurons were used.

Electrophysiological data were analyzed offline using Axograph X (RRID:SCR_014284; Axograph Scientific), Excel (Microsoft), and Prism (GraphPad).

### Calcium imaging

Cortical cultures grown on grid-bottomed dishes were infected with f(syn)-NES-jRCamP1b-WPRE-w, a lentivirus expressing jRCamP1b under the Synapsin promoter (Viral Core Facility, Charité – Universitätsmedizin), at 4 DIV. Images were acquired at 37°C and a CO_2_-controlled environment, using Nikon Spinning Disk Confocal CSU-X microscope with an air 20× Plan Apo objective lens (NA = 0.8), controlled via the NIS-Elements software (Nikon). Time-lapse images were collected at 5 Hz using an iXon3 DU-888 Ultra camera (Andor) and 561-nm excitation laser. In each dish, three fields of view with ∼10–20 cells were selected, and 2 min of spontaneous activity was measured twice, with 5-min interval between the runs. To study bicuculline-induced changes is network activity, bicuculline (30 μm) was manually pipetted into the cell culture dish immediately after the end of the second baseline activity recording. After a 30-s waiting period, two 2-min imaging sessions were recorded, separated by a 5-min waiting interval.

Time-lapse image analysis and automatic time-series event detection were accomplished with a custom-written script in OpenView software (Prof. Dr. Noam Ziv, Technion, Israel). In brief, ROIs were manually selected by placing boxes of 27 × 27 pixels over visually identified neuronal cell somas. Only active cells were included in analysis. Time series fluorescence values were converted into ΔF/F by calculating the ratio between the change in fluorescence signal intensity (δ F) and baseline fluorescence (F0). The custom-written algorithm identified the timestamps of calcium transient onset, which were then averaged per min to obtain the frequency of events.

### Experimental design and statistical analysis

GraphPad Prism was used to analyze and represent data. Schematic figures were created with BioRender.com. Statistical design, sample sizes, and tests for each experiment can be found in the figure legends. All figures represent data from at least three independent experiments (independent cultures). Unpaired and paired *t* tests and ANOVA Tukey's multiple comparison tests were used to evaluate statistical significance.

## Results

### hNR1 antibody increases spiking and bursting of cortical networks

**Figure 1.**
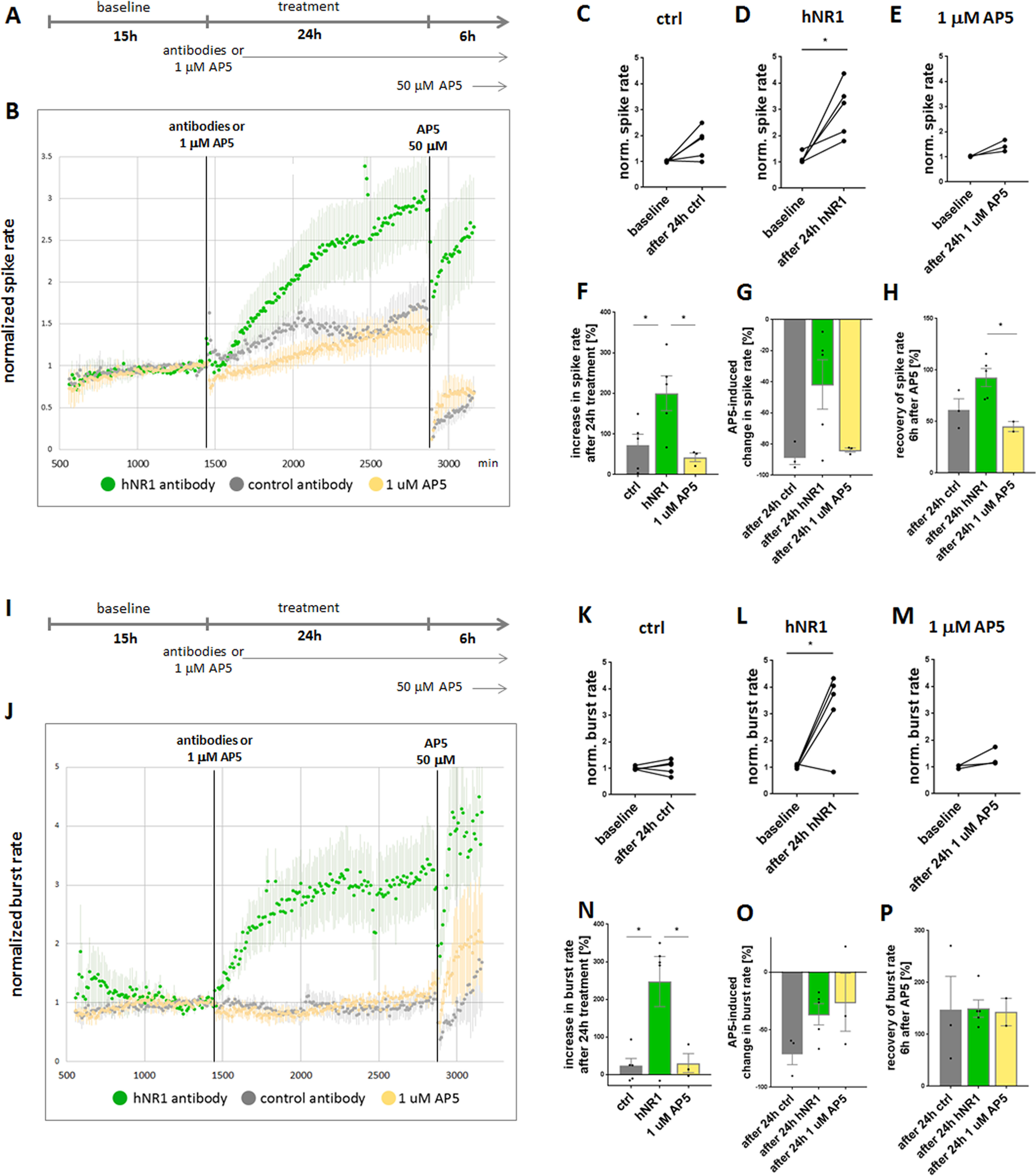
hNR1 antibody increases spiking and bursting activity of cortical cultures. ***A***, ***I***, Experimental design of MEA experiments. Baseline activity of cortical networks was recorded for 15 h, followed by addition of 1 μg/ml of hNR1 antibody, control (ctrl) antibody or 1 μm AP5 and 24-h recording of activity in the presence of antibodies/1 μm AP5. After 24 h of treatment, saturating concentration of AP5 (50 μm) was added to the network and another 6 h of activity were recorded. ***B***, Normalized spike rate of neuronal networks. Each data point represents a number of spikes recorded within a 10-min interval from all 59 electrodes and normalized to the last 3 h of baseline activity. Addition of hNR1 antibody (green data points), and to a much lesser degree control (ctrl, mGO53) antibody (gray data points) or AP5 at its IC_50_ concentration (1 μm; yellow data points), increase spiking of the network. hNR1 = 5 independent experiments, ctrl = 5 independent experiments, AP5 = 3 independent experiments. Error bars indicate SEM. ***C–E***, Within-dish comparison of normalized spike rate of the same network at the end of baseline recording (1 h) and after 24 h of treatment (1 h) tested by paired *t* test. ***C***, Baseline = 1.01 ± 0.01, after 24 h ctrl = 1.72 ± 0.27, 5, *p* = 0.07. ***D***, Baseline = 1,12 ± 0.47, after 24-h hNR1 = 3.02 ± 0.47, *p* = 0.02. ***E***, Baseline = 1.02 ± 0.01, after 24 h 1 μm AP5 = 1.43 ± 0.13, *p* = 0.09. ***F–H***, Percentage increase in normalized spike rate at the end (10 min) or beginning (10 min) of respective treatment. Each data point represents individual network (MEA dish). Percentage increase in spike rate: (***F***) at the end of baseline compared with end of antibody treatment, ctrl = 71.71 ± 27.46%; hNR1 = 200.5 ± 42.96%, 1 μm AP5 = 42.4 ± 10.76%, ctrl versus hNR1 *p* = 0.045, hNR1 versus 1 μm AP5 *p* = 0.034; (***G***) at the end of antibody treatment compared with beginning of 50 μm AP5, ctrl = −94.96 ± 5.03%; hNR1 = −41.69 ± 15.88%; 1 μm AP5 = −86.51 ± 1.33%, ctrl versus hNR1 *p* = 0.085; (***H***) at the end of antibody treatment compared with recovery 6 h after addition of 50 μm AP5, ctrl = 61.34 ± 10.65%; hNR1 = 92.65 ± 8.8%; 1 μm AP5 = 45.04 ± 4.88%, hNR1 versus 1 μm AP5 *p* = 0.038. ***J***, Normalized burst rate of neuronal networks. Each data point represents a number of bursts recorded within a 10-min interval, normalized to the last 3 h of baseline. Network bursting increases in the presence of hNR1 antibody, but not of control antibody nor 1 μm AP5. ***K–M***, Within-dish comparison of normalized burst rates of the same networks at the end of baseline recording (1 h) and after 24 h of treatment (1 h) tested by paired *t* test. ***K***, Baseline = 1 ± 0.03; after 24 h ctrl = 1.05 ± 0.12, *p* = 0.72. ***L***, Baseline = 1.04 ± 0.03; after 24-h hNR1 = 3.22 ± 0.63, *p* = 0.027. ***M***, Baseline = 1 ± 0.04; after 24 h 1 μm AP5 = 1.36 ± 0.2, *p* = 0.2. ***N–P***, Percentage increase in normalized burst rate at the end (10 min) or beginning (10 min) of respective treatments. Percentage increase in burst rate: (***N***) at the end of baseline compared with end of antibody treatment, ctrl = 23.31 ± 19.47%; hNR1 = 247.5 ± 66.75%; 1 μm AP5 = 30.14 ± 25.57%, ctrl versus hNR1 *p* = 0.01, hNR1 versus 1 μm AP5 *p* = 0.03; (***O***) end of antibody treatment compared with beginning of 50 μm AP5, ctrl = −70.46 ± 9.9%; hNR1 = −36.58 ± 25.24%; 1 μm AP5 = −26.05 ± 25.24%; (***P***) end of antibody treatment compared with recovery 6 h after addition of 50 μm AP5, ctrl = 147 ± 64.39%; hNR1 = 149.6 ± 16.73%; 1 μm AP5 = 143 ± 26.56%. Error bars indicate SEM. Paired *t* test (***C–E***, ***K–M***) or ANOVA Tukey's multiple comparison test (***F–H***, ***N–P***) were used to evaluate statistical significance. *p < 0.05.

Previous studies have shown that human anti-NMDAR antibodies affect NMDAR function in hippocampal neurons, helping to explain potential mechanistic causes for cognitive impairment in patients with autoimmune NMDAR encephalitis (Hughes et al., 2010; Mikasova et al., 2012; Würdemann et al., 2016). However, other symptoms centered in cortical circuits, such as psychiatric manifestations, catatonia, coma and epilepsy, have yet to be explored. We were thus interested in addressing the question of whether these antibodies can also affect cortical network function and if so, what is their functional impact? To explore these fundamental issues, we took advantage of a patient-derived monoclonal antibody #003-102 targeting the NR1 subunit of NMDAR (hNR1; Kreye et al., 2016) and tested its effect on primary cortical neuron cultures. We began with examining effects of hNR1 antibody on neuronal network activity by growing cortical neurons on multielectrode array (MEA) dishes for 11–14 d. This approach allows a continuous and simultaneous monitoring of activity from dozens of neurons recorded by 59 electrodes. Initially, baseline spiking activity of the neuronal network was recorded for 15 h, afterward 1 µg/ml of hNR1 or control antibody (ctrl; mGO53; Wardemann et al., 2003) were added and the spiking activity was traced for another 24 h (Fig. 1*A*,*I*). Remarkably, we observed a dramatic change in network spiking activity in the presence of hNR1 antibody that gradually increased over the period of 24 h of antibody treatment, reaching a highly active state (baseline = 1.12 ± 0.47, after 24-h hNR1 = 3.02 ± 0.47, paired *t* test, *p* = 0.02; Fig. 1*B*,*D*). The effect of the control antibody was more limited, eliciting a modest, but not significant, change in network spiking activity during this period (normalized baseline = 1.01 ± 0.01, after 24 h ctrl = 1.72 ± 0.27, 5, paired *t* test, *p* = 0.07; Fig. 1*B*,*C*).

The observed increase in spiking caused by the hNR1 antibody is both surprising and counterintuitive, as earlier studies demonstrated that active neuronal networks commonly respond to blockage of NMDARs, by NMDAR antagonists like AP5 or ketamine, with a rapid decrease of their spiking activity (Emnett et al., 2013; Teppola et al., 2018, 2019), which slowly recovers over hours, because of various slow adaptive processes (Kaufman et al., 2014). Conceptually, this difference could be because of the fact that the hNR1 antibody only blocks ∼50% of the whole-cell NMDAR currents in cultured hippocampal neurons (Hughes et al., 2010; Kreye et al., 2016), while saturating concentrations of AP5 and ketamine block all active NMDARs. We therefore hypothesized that the hNR1 antibody-induced increase in spiking activity could be an adaptive response of the network to an incomplete block of NMDARs. To test this hypothesis, we conducted similar experiments by adding 1 μm of the NMDAR antagonist AP5 (1 μm being the IC_50_ for NMDARs) instead of antibody, to block 50% of the surface NMDARs. Here, the presence of 1 μm AP5 was associated with a modest, yet nonsignificant, increase in spiking activity over 24 h (baseline = 1.02 ± 0.01, after 24 h 1 μm AP5 = 1.43 ± 0.13, paired *t* test, *p* = 0.09; Fig. 1*B*,*E*, yellow data points). As the magnitude of this change was just a fraction of that elicited by hNR1 antibody (∼43% vs ∼300%, respectively; Fig. 1*B,D-F*), it is possible that the underlying mechanism of the latter could be different, e.g., by affecting a subpopulation of receptors or cell types. Of note, AP5 is expected to act more quickly than the hNR1 antibody, yet as both conditions involved chronic multihour treatments, the observed outcomes are anticipated to be because of more slow adapting processes by both manipulations.

An interesting consequential question is whether networks treated with hNR1 antibody or AP5 are still responsive to pharmacological manipulations of NMDARs. This was examined by adding saturating concentrations of AP5 (50 μm) at the end of the 24-h treatment with hNR1, control antibody or 1 μm AP5 (Fig. 1*A*). Interestingly, networks treated with the control antibody or 1 μm AP5 remained responsive to high concentration of AP5, as their spiking rapidly decreased (95% and 87% reduction, respectively; Fig. 1*B*,*G*). Furthermore, spiking only partially recovered to ∼60% of their initial value 6 h later (ctrl = 61%, AP5 = 45%; Fig. 1*F*). In contrast, the spiking activity of cortical cultures treated with hNR1 antibody only partially dropped because of AP5 treatment (42% reduction; Fig. 1*G*) and quickly recovered to 93% of the activity levels preceding AP5 treatment (Fig. 1*H*). These remained nearly fourfold higher than those observed in networks exposed to the control antibody or 1 μm AP5. Together, these data indicate that the addition of hNR1 antibody to cortical cultures renders the network largely insensitive to NMDAR blockage with AP5.

Subsequently, we asked whether other parameters crucial for behavior of neuronal network, such as bursting, number of spikes per burst and network synchrony, are also affected by hNR1 antibody treatment. While synchrony and number of spikes per burst were not different between the groups (data not shown), we observed a dramatic increase in network bursting activity because of hNR1 antibody treatment (baseline = 1.04 ± 0.03; after 24-h hNR1 = 3.22 ± 0.63, paired *t* test, *p* = 0.027; Fig. 1*J*,*L*). Burst rate of both control antibody (baseline = 1 ± 0.03; after 24 h ctrl = 1.05 ± 0.12, paired *t* test, *p* = 0.72) and 1 μm AP5 (baseline = 1 ± 0.04; after 24 h 1 μm AP5 = 1.36 ± 0.2, paired *t* test, *p* = 0.2) treated networks was not changed (Fig. 1*J–N*). Intriguingly, the hNR1 antibody-induced increase in burst rate seemed to have a more rapid onset than the increase in neuronal spiking (Fig. 1*B*,*J*), reaching a plateau after ∼7 h of hNR1 antibody treatment (Fig. 1*J*). This phenomenon of elevated burst rates is a characteristic of networks in which inhibition has been removed (Chen et al., 2012). Interestingly, high concentrations of AP5 had a smaller effect on bursting activity than on spiking. Moreover, its immediate effects (Fig. 1*O*), or recovery of burst rate 6 h later (Fig. 1*P*), were not different between the treatments.

To summarize, these experiments reveal two unexpected effects of hNR1 antibody on cortical neurons: (1) substantial elevations in cortical network spiking and bursting activity and (2) a partial loss of responsiveness to NMDAR blockage by AP5, a situation that could conceivably both adversely affect network function and drive epileptic brain activity in patients.

### hNR1 antibody impairs NMDA-mediated Npas4 expression in cortical cultures

**Figure 2.**
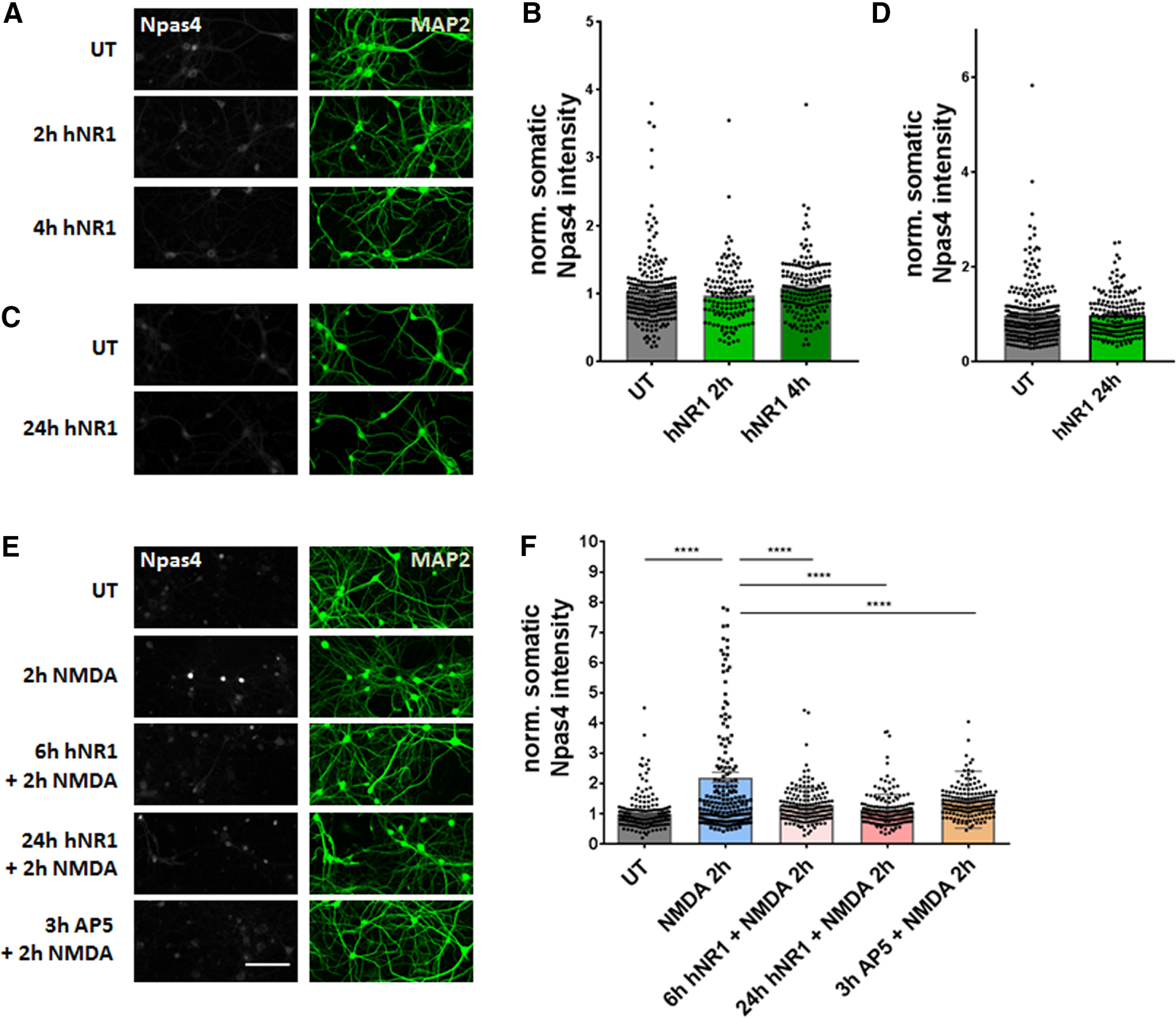
hNR1 antibody dysregulates NMDA-induced expression of Npas4, a transcription factor regulating network activity. ***A–D***, Cortical neuronal cultures 14–16 DIV were incubated with 1 µg/ml of hNR1 antibody for 2, 4, or 24 h, then fixed and stained with anti-Npas4 antibody to measure intensity of somatic Npas4 expression. Neither short-term (2, 4 h, ***A***) nor long-term (24 h, ***C***) treatment with hNR1 induced somatic Npas4 expression when compared to untreated (UT) condition. ***B***, ***D***, Normalized intensity of somatic Npas4 signal from three independent experiments: (***B***) UT = 1 ± 0.03, *n* = 218 neurons; 2-h hNR1 = 0.97 ± 0.04, *n* = 133 neurons; 4-h hNR1 = 1.08 ± 0.03, *n* = 168 neurons; (***D***) UT = 1 ± 0.04, *n* = 280 neurons; 24-h hNR1 = 0.98 ± 0.03, *n* = 183 neurons. ***E***, Stimulation of neurons with 2 μm NMDA for 2 h induces activity-driven increase of Npas4 expression compared with untreated (UT) condition. Six- or 24-h pretreatment with hNR1 antibody (1 µg/ml), as well as 3-h pretreatment with NMDAR antagonist AP5 (50 μm), blocks NMDA-induced expression of Npas4. ***F***, Normalized intensity of somatic Npas4 signal from three independent experiments: UT = 1 ± 0.03, *n* = 235 neurons; NMDA = 2.21 ± 0.17, *n* = 197 neurons; 6 h NR1 + NMDA = 1.28 ± 0.04, *n* = 172 neurons; 24-h hNR1 – NMDA = 1.13 ± 0.03, *n* = 179 neurons; 3 h AP5 + NMDA = 1.47 ± 0.07, *n* = 166 neurons. Error bars indicate SEM. Scale bar: 100 µm. ANOVA Tukey's multiple comparison test (***B***, ***F***) or paired *t* test (***D***) were used to evaluate statistical significance. ****p < 0.0001.

Neuronal networks are complex systems whose function depends on the proper function of various neuronal cell types and regulatory mechanisms, allowing them to maintain their activity within a narrow range. Normally under increasing activity conditions, calcium influx through NMDARs signals to the cell nucleus and triggers an adaptive change in neuronal gene expression profiles, resulting in a homeostatic restoration of activity levels (Hardingham and Bading, 2003; Flavell and Greenberg, 2008). One such activity-dependent transcription factor, whose expression is partially triggered by calcium influx through NMDARs, is Neuronal PAS domain protein 4 (Npas4; Bloodgood et al., 2013; Spiegel et al., 2014). It was thus of interest to know whether this transcriptional program still operates following the addition of hNR1 antibody. Specifically, we asked whether the hNR1 antibody-induced increase in network activity (Fig. 1) was associated with a corresponding increase in Npas4 expression (Fig. 2). Surprisingly, treating cortical cultures with hNR1 antibody for 2, 4, or 24 h did not induce the expression of Npas4, as measured by changes in fluorescence intensity of somatic Npas4 in cortical cultured neurons (Fig. 2*A–D*). These data indicate that a Npas4-mediated homeostatic mechanism is not active and/or cannot be engaged in the presence of the hNR1 antibody. As a more direct test of the latter, we asked whether hNR1 antibody disrupts NMDA-mediated regulation of Npas4 expression. As expected under control conditions, the addition of NMDA to cortical cultures induced significant Npas4 expression (untreated UT = 1 ± 0.03, NMDA = 2.21 ± 0.17, *p* < 0.0001; Fig. 2*E*, first and second panels, *F*). However, the pretreatment with hNR1 antibody for 6 or 24 h blocked the increase in somatic Npas4 expression, similarly to networks pretreated with AP5 (6 h NR1 + NMDA = 1.28 ± 0.04, 24-h hNR1 + NMDA = 1.13 ± 0.03, 3 h AP5 + NMDA = 1.47 ± 0.07, *p* < 0.0001; Fig. 2*E*,*F*). These data suggest that hNR1 antibody interferes with the capacity of cortical networks to decrease their activity through NMDA/Npas4 regulated gene expression, an observation that could help explain why the hNR1 antibody-induced hyperactivity is not downregulated to homeostatic levels over time. However, less clear is whether the dysregulation of NMDA/Npas4 regulated gene expression can explain why hNR1 antibody treatment increases network activity in the first place, or whether other mechanisms are at play that more directly regulate excitatory/inhibitory balance in these networks.

### hNR1 antibody disinhibits cortical networks by specifically impairing output of inhibitory neurons

**Figure 3.**
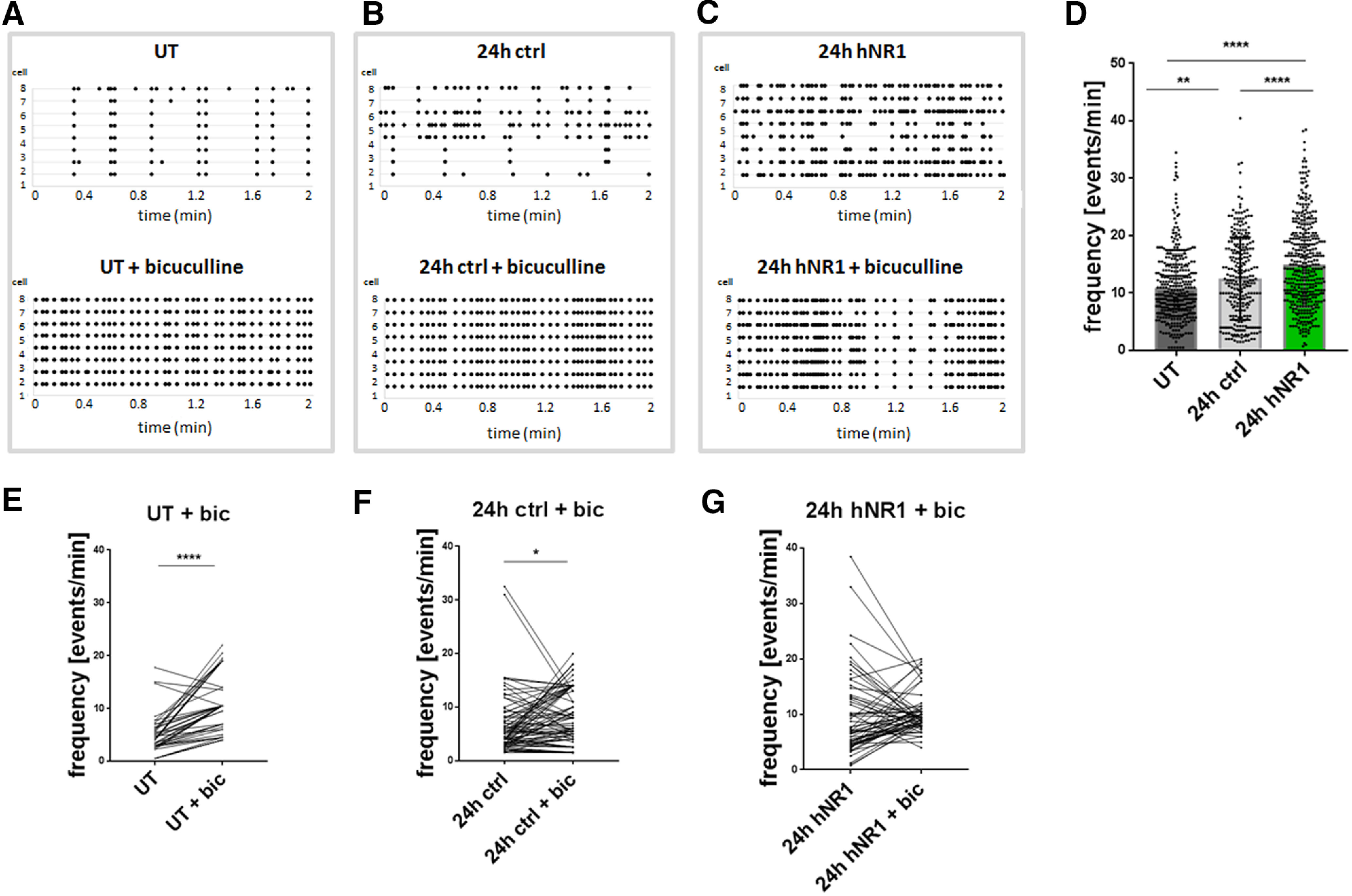
Calcium imaging reveals that networks treated with hNR1 antibody cannot be further disinhibited by bicuculline. ***A–C***, Representative raster plots of calcium events recorded from neuronal somas (neuron ID on the *y*-axis) expressing RCaMP in untreated condition (UT) (***A***, upper panel), after 24 h of control antibody (***B***, upper panel) or 24 h of hNR1 antibody (***C***, upper panel) treatment. ***A–C***, Lower panels: representative raster plots of calcium events of the same neurons as in upper panels following addition of bicuculline (bic, 30 μm) at the end of respective treatment. ***D***, Frequency of somatic calcium events in untreated condition, after 24 h of hNR1 or control antibodies treatment (***A–C***, upper panels); UT = 10.89 ± 0.31, *n* = 366 cells, ctrl = 12.55 ± 0.43, *n* = 277 cells, hNR1 = 14.99 ± 0.37, *n* = 391 cells, 6 independent experiments, **p = 0.007, ****p < 0.0001. ***E–G***, Within-cell comparison of the frequency of calcium events in the same cells at the end of 24-h treatment and after addition of bicuculline, tested by paired *t* test from three independent experiments, reveals that bicuculline disinhibits the networks in untreated condition (***D***) and after control antibodies treatment (***E***) but not after hNR1 (***F***) treatment. Quantification of calcium events: (***E***) UT = 4.83 ± 0,51, UT + Bic = 10.87 ± 0,81, mean of difference = 6.05 ± 0.75, *n* = 47 neurons, *p* < 0.0001; (***F***) 24 h ctrl = 6.84 ± 0.0.74, 24 h ctrl + Bic = 8.61 ± 0,59, mean of difference = 1.76 ± 0.82, *n* = 80 neurons, *p* = 0.035; (***G***) 24-h hNR1 = 9.88 ± 0.88, 24-h hNR1 + Bic = 10.24 ± 0.45, mean of difference = 0.57 ± 0.9, *n* = 62 neurons, *p* = 0.53. Error bars indicate SEM. ANOVA Tukey's multiple comparison test (***D***) and paired *t* test (***E–G***) were used to evaluate statistical significance. *p < 0.05, **p < 0.01, ****p < 0.0001.

Proper function of cortical networks is determined by a fine balance between excitation and inhibition. Previous studies have shown that hypofunction of NMDARs can reduce inhibition and disinhibit cortical networks (Homayoun and Moghaddam, 2007; Zhang et al., 2008). It thus seemed feasible that the hNR1 antibody-induced hyperactivity is a result of network disinhibition. To test this hypothesis, we designed a set of calcium imaging and electrophysiological experiments. For calcium imaging, neurons were initially infected at 4 DIV with a lentivirus expressing RCaMP (Dana et al., 2016), a genetically encoded calcium indicator, and allowed to grow for 10 more days (14 DIV) before antibody treatment. Cortical networks of neurons were then subjected to live imaging 24 h after treatment with hNR1 or control antibodies. Subsequently, activity levels were measured by detecting somatic calcium transients of individual cells and compared with untreated cultures. Similar to MEA recordings (Fig. 1), in cortical cultures treated with the hNR1 antibody the frequency of somatic calcium events dramatically increased, as compared with both untreated networks and those treated with control antibody (UT = 10.89 ± 0.31, ctrl = 12.55 ± 0.43, hNR1 = 14.99 ± 0.37, *p* < 0.0001; Fig. 3*A–C*, upper panels, *D*). Interestingly, in this experimental setup the control antibody (ctrl) also increased the frequency of calcium events compared with untreated cultures (*p* = 0.007), although to a lesser extent than the hNR1 antibody (ctrl: 15%, hNR1: 38% increase; Fig. 3*D*). These latter data suggest that this antibody is not without its effects, although the mechanism and its antigen remain unknown.

Given the high spiking and bursting frequency of cortical cultures treated with hNR1 antibody, it was of interest to explore whether such cultures could be further disinhibited, perhaps by disengaging the GABAergic system. This was accomplished by adding bicuculline (30 μm), a GABA_A_R antagonist, at the end of 24-h treatment with antibodies and re-imaging activity of the same neurons for a within-cell comparison (Fig. 3*A–F*). As expected, in untreated and control antibody treated conditions, bicuculline increased the frequency of calcium events (paired *t* test, *p* < 0.0001, *p* = 0.035, respectively; Fig. 3*E*,*F*). Strikingly, bicuculline failed to further increase the frequency of calcium events in cultures treated with the hNR1 antibody (paired *t* test, *p* = 0.53; Fig. 3*G*). These data imply that critical features of the GABAergic system and/or Ca^2+^-activated potassium channels, also known to be inhibited by bicuculline (Khawaled et al., 1999; Johnston, 2013), could contribute to antibody-induced hyperexcitability.

**Figure 4.**
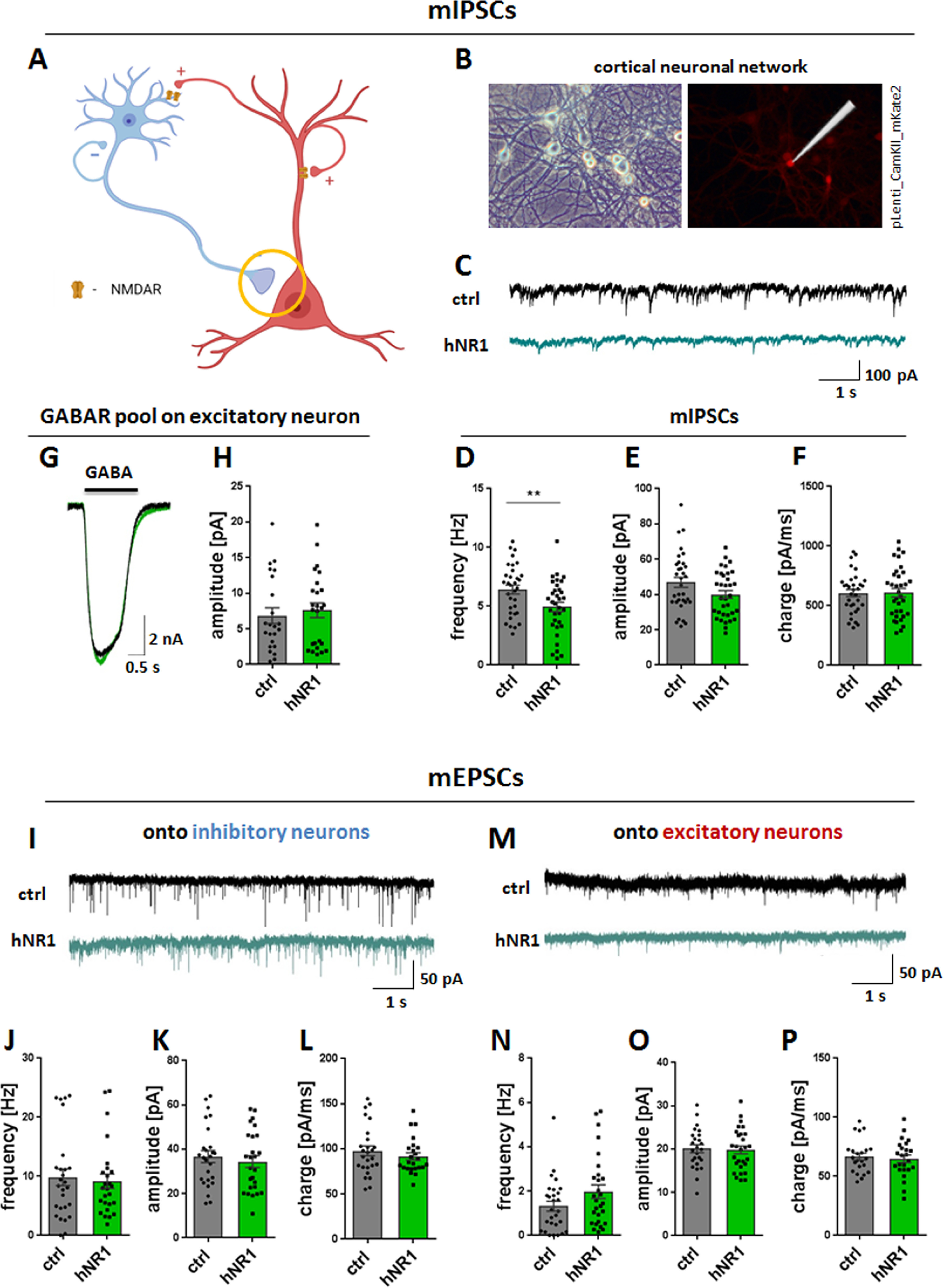
hNR1 antibody decreases inhibitory drive onto excitatory neurons in cortical circuits but does not affect excitatory drive. ***A–H***, Inhibitory drive onto excitatory neurons was measured by mIPSCs recorded from excitatory neurons in cortical neuronal networks. ***A***, Schematic model of a simplified neuronal network composed of excitatory pyramidal neurons (red) and inhibitory neurons (blue), forming different types of synapses onto each other. Inhibitory synapse onto excitatory neuron is marked with a circle as a synapse of interest measured in this experiment. ***B***, mIPSCs were recorded from excitatory neurons identified by expression of mKate2 expressed under excitatory neuron CamKII promoter. Left panel, BF image of representative cortical neuronal network. Right panel, Excitatory neuron identified by mKate2 fluorescent signal. ***C***, Representative traces of mIPSCs recorded in a whole-cell patch-clamp configuration from excitatory neurons after 24 h of treatment with control (ctrl, upper panel) or hNR1 (lower panel) antibodies. Treatment with hNR1 antibody decreases frequency of mIPSCs onto excitatory neurons (***D***) and has tendency to decrease their amplitude (***E***), without affecting their charge (***F***). ***D–F***, Quantification of mIPSCs parameters from three independent experiments, ctrl *n* = 33 neurons, hNR1 *n* = 34 neurons: (***D***) mIPSCs frequency: ctrl = 6.42 ± 0.37, hNR1 = 4.94 ± 0.4, *p* = 0.009; (***E***) mIPSCs amplitude: ctrl = 46.89 ± 2.89, hNR1 = 40.01 ± 2.25, *p* = 0.06; (***F***) mIPSCs charge: ctrl = 606.5 ± 29.72, hNR1 = 610 ± 36.37. ***G***, ***H***, hNR1 antibody treatment does not affect levels of GABA receptors on the postsynaptic excitatory neurons from which mIPSCs were recorded. ***G***, Representative traces of GABA currents recorded from excitatory neurons, evoked by 1-s pulse application of 5 μm GABA. ***H***, Quantification of GABA current amplitude presented in (***F***): ctrl = 6.84 ± 1.1, *n* = 23 neurons, hNR1 = 7.6 ± 1.04, *n* = 7.6 ± 1.04, *n* = 25 neurons. ***I–P***, hNR1 antibody does not affect excitatory miniature transmission (mEPSCs) onto neither inhibitory (***I–L***) nor excitatory (***M–P***) neurons. ***I***, ***M***, Representative traces of mEPSCs recorded from inhibitory (***I***) or excitatory (***M***) neurons. Quantification of mEPSCs parameters from three independent experiments: (***J***) mEPSCs frequency: ctrl = 9.83 ± 1.41, *n* = 27 neurons, hNR1 = 9.17 ± 1.2, *n* = 26 neurons; (***K***) mEPSCs amplitude: ctrl = 36.7 ± 2.71, *n* = 26 neurons, hNR1 = 34.41 ± 2.68, *n* = 26 neurons; (***L***) mEPSCs charge: 97.83 ± 5.47, *n* = 26 neurons, hNR1 = 91.52 ± 4.02, *n* = 24 neurons; (***N***) mEPSCs frequency: ctrl = 1.32 ± 0.23, *n* = 28 neurons, hNR1 = 1.98 ± 0.21, *n* = 28 neurons; (***O***) mEPSCs amplitude: ctrl = 20.09 ± 0.93, *n* = 25 neurons, hNR1 = 19.84 ± 0.92, *n* = 28 neurons; (***P***) mEPSCs charge: ctrl = 66.76 ± 2.96, *n* = 23 neurons, hNR1 = 64.86 ± 3.22, *n* = 24 neurons). Error bars indicate SEM. Unpaired *t* test was used to evaluate statistical significance. **p < 0.01.

As the GABAergic system is a known key regulator of excitatory and inhibitory balance, we designed several experiments to further explore possible inhibitory dysfunction in the context of hNR1 antibody-disrupted NMDAR signaling. Interestingly, studies on the “glutamatergic hypothesis of schizophrenia” suggest that hypofunction of inhibitory drive onto excitatory pyramidal cells leads to disinhibition of cortical networks (Homayoun and Moghaddam, 2007), producing psychotic symptoms similar to those observed in patients with NMDAR encephalitis. We therefore asked whether the inhibitory drive onto excitatory neurons is also altered in cortical networks because of hNR1 antibody. Here, we performed a set of patch-clamp electrophysiology experiments to record miniature inhibitory post-synaptic currents (mIPSCs) from excitatory neurons in cortical cultures (Fig. 4*A*). Excitatory neurons were identified by infecting WT neurons with pLenti_CamKII_mKate2, a lentivirus expressing mKate2 under the glutamatergic specific CamKII promoter, at DIV2–DIV4 (Fig. 4*B*). At 15–18 DIV, we recorded mIPSCs from these neurons, after 24-h treatment with either hNR1 or control antibody. We observed that hNR1 antibody treatment significantly decreased mIPSCs frequency (ctrl = 6.42 ± 0.37, hNR1 = 4.94 ± 0.4, unpaired *t* test, *p* = 0.009) and had a tendency to decrease mIPSCs amplitude (ctrl = 46.89 ± 2.89, hNR1 = 40.01 ± 2.25, unpaired *t* test, *p* = 0.06) compared to treatment with control antibodies (Fig. 4*C–E*) without affecting charge (Fig. 4*F*). These data indicate that 24-h hNR1 antibody treatment indeed decreased inhibitory drive onto excitatory neurons. To test whether this is caused by an overall reduction of surface GABAR function on postsynaptic excitatory neurons, we measured currents induced by bath application of GABA (5 μm) using a fast-flow system. However, these GABA currents were similar between control and hNR1 antibody treated cultures (Fig. 4*G*,*H*), indicating that the total pools of GABA_A_R on excitatory neurons are not altered.

As network activity is sensitive to both excitation and inhibition, we next explored whether the excitatory drive in neuronal cultures was also altered by hNR1 antibody. This was accomplished by recording miniature excitatory post-synaptic currents (mEPSCs) in the presence of antibodies from both excitatory and inhibitory cortical neurons. Interestingly, the frequency, amplitude, and charge of mEPSCs onto either cell type were not affected by hNR1 antibody treatment (Fig. 4*I–P*). These data indicate that hNR1 antibody specifically reduces the inhibitory drive onto excitatory neurons in a cortical network, which could be causal for its overall disinhibition.

### hNR1 antibody specifically impairs the function of inhibitory and not excitatory autapses

**Figure 5.**
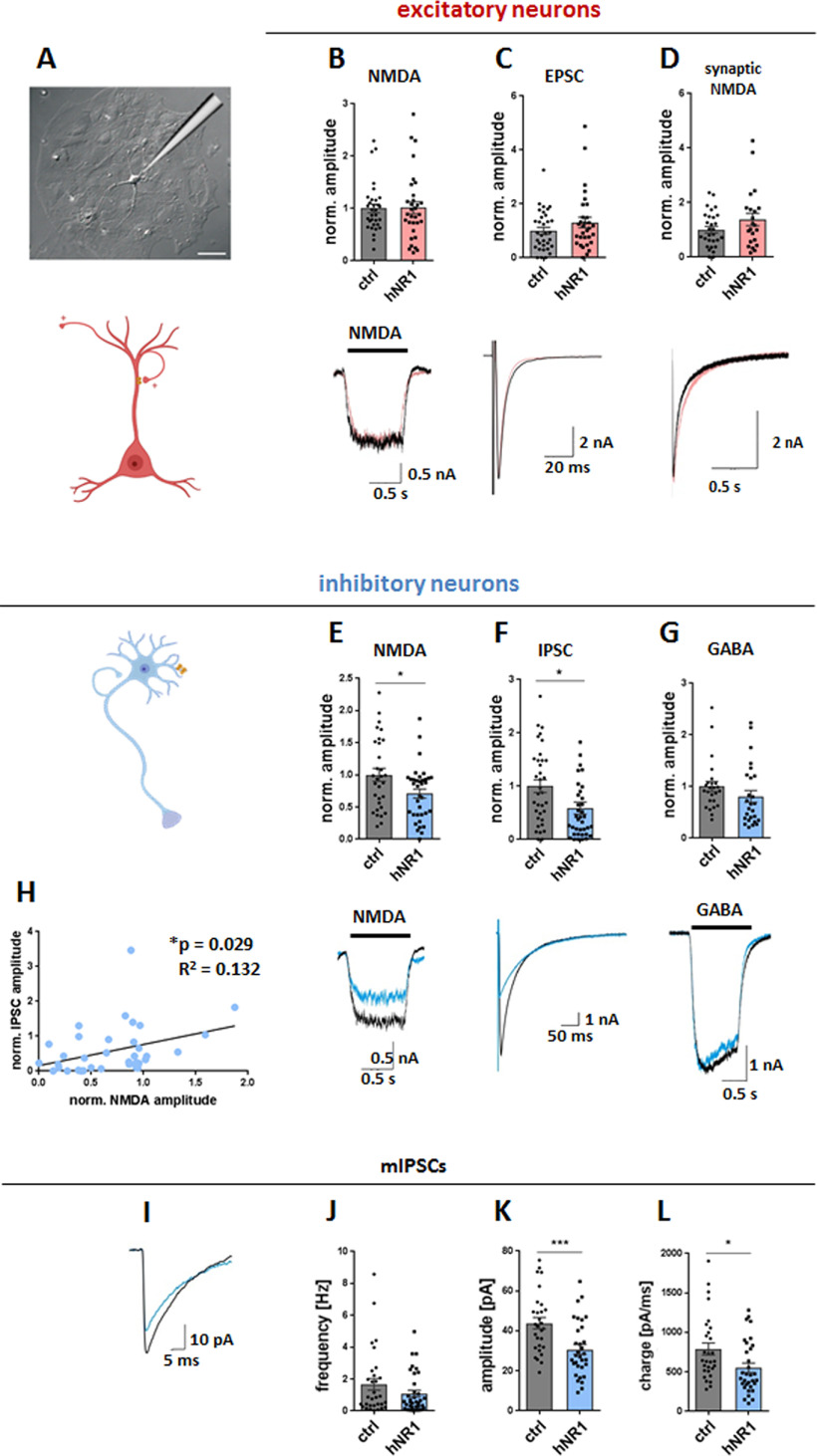
hNR1 antibody impairs function of cortical inhibitory, but not excitatory neurons, in a cell-autonomous manner. ***A***, Example image of a single cortical neuron grown on an astrocytic island in autaptic culture and a schematic representation of a patch pipette approaching the neuron. ***B–D***, Lower panels, representative traces of (***B***) whole-cell NMDA currents evoked by 1s bath application of NMDA (10 μm), (***C***) evoked EPSCs, (***D***) evoked synaptic NMDA currents in excitatory neurons after 24-h treatment with control (ctrl) or hNR1 antibodies. Upper panels, normalized current amplitudes from 4 independent experiments on excitatory neurons: (***B***) ctrl = 1 ± 0.08, *n* = 32 neurons, hNR1 = 1.01 ± 0.11, *n* = 32 neurons; (***C***) ctrl = 1 ± 0.13, *n* = 33 neurons, hNR1 = 1.31 ± 0.19, *n* = 31 neurons; and (***D***) ctrl = 1 ± 0.12, *n* = 29 neurons, hNR1 = 1.38 ± 0.23, *n* = 22 neurons. ***E–G***, Lower panels, representative traces of (***E***) whole-cell NMDA currents, (***F***) evoked IPSCs, (***G***) whole-cell GABA currents evoked by 1s bath application of GABA (5 μm) of inhibitory neurons after 24-h treatment with control or hNR1 antibodies. Upper panels, normalized current amplitudes from 4 independent experiments: (***E***) ctrl = 1 ± 0.1, *n* = 31 neurons, hNR1 = 0.71 ± 0.07, *n* = 36 neurons, *p* = 0.018; (***F***) ctrl = 1 ± 0.12, *n* = 35 neurons, hNR1 = 0.59 ± 0.11, *n* = 37 neurons, *p* = 0.014; (***G***) ctrl = 1 ± 0.1, *n* = 25 neurons, hNR1 = 0.81 ± 0.11, *n* = 26 neurons. ***H***, Correlation between normalized IPSC and NMDA current amplitudes of individual inhibitory neurons. Linear regression: *R*^2^ = 0.132, *p* = 0.029. ***I–L***, Analysis of mIPSCs recorded from autaptic inhibitory neurons after 24 h of control or hNR1 antibodies treatment, 4 independent experiments. ***I***, Representative traces of mIPSCs. ***J***, mIPSCs frequency: ctrl = 1.66 ± 0.37, *n* = 31 neurons, hNR1 = 1.08 ± 0.21, *n* = 35 neurons. ***K***, mIPSCs amplitude: ctrl = 43.87 ± 2.81, *n* = 29 neurons, hNR1 = 30.72 ± 2.46, *n* = 32 neurons, *p* = 0.0008. ***L***, mIPSCs charge: ctrl = 789.7 ± 76.58, *n* = 29 neurons, hNR1 = 551.9 ± 58.94, *n* = 32 neurons, *p* = 0.016. Error bars indicate SEM. Unpaired *t* test (***B–D***, ***E–G***, ***J–L***) and linear regression (***H***) were used to evaluate statistical significance. *p < 0.05, ***p < 0.001.

As we observed a decrease in overall inhibition and specific changes in inhibitory neuron output, we next examined the selective effects of the hNR1 antibody on inhibitory cortical neurons in more detail. Hence, we performed patch-clamp electrophysiological recordings in an autaptic system, where single neurons are grown on an astrocytic microislands, allowing the study of cell-autonomous effects on either inhibitory or excitatory neurons (Fig. 5*A*). To easily distinguish between these two neuronal populations, we employed GAD67-GFP mice, in which inhibitory neurons expressing GAD67 are fluorescently labeled (Tamamaki et al., 2003). Previous groups reported no changes in electrophysiological properties of these neurons when compared with WT neurons (Chang et al., 2014). In initial experiments, we examined whether the addition of hNR1 or control antibodies (24 h) affect the total surface expression of the 3 main groups of ionotropic receptors responsible for synaptic transmission, by acutely bath-applying NMDA (10 μm), GABA (5 μm), or kainate (20 μm) in 1-s pulses and comparing responses of inhibitory and excitatory cells. Intriguingly, we observed that in autaptic cultures of cortical neurons, hNR1 antibody treatment did not affect whole-cell NMDA currents on excitatory neurons (ctrl = 1 ± 0.08, hNR1 = 1.01 ± 0.11, unpaired *t* test, *p* = 0.92; Fig. 5*B*), yet it selectively decreased whole-cell NMDA currents on inhibitory neurons (ctrl = 1 ± 0.1, hNR1 = 0.71 ± 0.07, unpaired *t* test, *p* = 0.018; Fig. 5*E*). At the same time, total GABA-specific and kainate-specific currents in either cell type remained unchanged (Fig. 5*G*, data not shown). Next, we measured evoked synaptic responses by inducing an action potential in these cells, by a brief somatic depolarization of the neuron, and recording of the postsynaptic current that followed a few milliseconds afterward. Intriguingly, on inhibitory neurons, synaptic inhibitory transmission (IPSCs) was significantly reduced by ∼41% (ctrl = 1 ± 0.12, hNR1 = 0.59 ± 0.11, *p* = 0.01; Fig. 5*F*), whereas synaptic excitatory transmission (EPSCs; ctrl = 1 ± 0.13, hNR1 = 1.31 ± 0.19, unpaired *t* test, *p* = 0.19; Fig. 5*C*) and pharmacologically isolated synaptic NMDA currents (ctrl = 1 ± 0.12, hNR1 = 1.38 ± 0.23, unpaired *t* test, *p* = 0.13; Fig. 5*D*) on excitatory neurons remained unchanged (Fig. 5*C*,*D*). What is more, on inhibitory neurons, the reduction of NMDA currents and the amplitude of IPSCs seemed to go hand in hand, as there was a significant correlation between these two parameters among inhibitory cells (linear regression, *R*^2^ = 0.132, *p* = 0.029; Fig. 5*H*). These data suggest that in autaptic cortical neurons, hNR1 antibody treatment primarily affects the function of NMDARs on inhibitory neurons, which in turn reduces inhibitory synaptic output by these cells. To explore the mechanism further, we examined the impact of hNR1 antibody on the spontaneous release of GABA from inhibitory neurons by measuring mIPSCs in these autaptic cultures. Here, we observed a significant decrease in mIPSCs amplitude (Fig. 5*K*; ctrl = 43.87 ± 2.81, hNR1 = 30.72 ± 2.46, unpaired *t* test, *p* = 0.0008) and charge (Fig. 5*L*; ctrl = 789.7 ± 76.58, hNR1 = 551.9 ± 58.94, unpaired *t* test, *p* = 0.016). Similar to neuronal network experiments (Fig. 4*G*,*H*), we observed no differences in responses after bath application of GABA between autaptic neurons treated with control or hNR1 antibodies (Fig. 5*G*). Together, these data indicate that the hNR1 antibody specifically impairs NMDAR– and synaptic transmission of cortical inhibitory, but not excitatory neurons, in a cell-autonomous manner.

Importantly, as we observed a modest effect of the control antibody on neuronal network activity (Fig. 3), we evaluated whether it also exerts any significant effects in autaptic cultures. Here, no differences between untreated and control antibody treated cultures were observed in any of reported measures (whole-cell NMDA, GABA, and kainate currents, synaptic NMDA currents and EPSCs/IPSCs) in neither excitatory nor inhibitory neurons (data not shown). These data suggest that the control antibody generally has no direct effect on synaptic function in the autaptic system.

### hNR1 antibody exerts different effects in cortical versus hippocampal neurons

**Figure 6.**
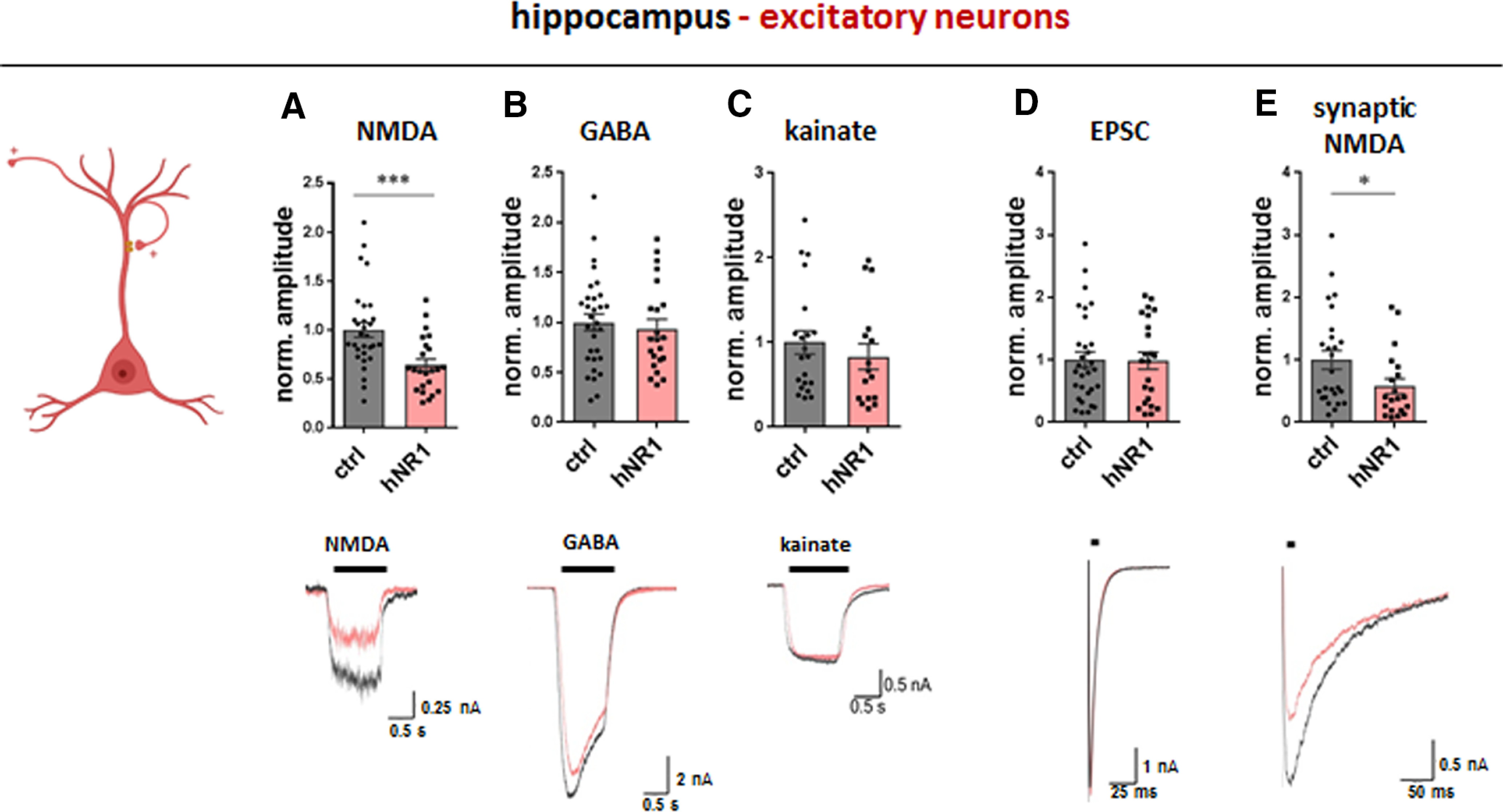
hNR1 antibody impairs NMDA currents of excitatory hippocampal neurons. In hippocampal autaptic cultures, 24-h treatment with hNR1 antibody (1 µg/ml) selectively decreases whole-cell (***A***) and synaptic (***E***) NMDA currents of excitatory neurons, when compared to treatment with control (ctrl) antibody. ***A–C***, Whole-cell receptor currents were evoked by 1s bath application of (***A***) NMDA (10 μm), (***B***) GABA (30 μm), or (***C***) kainate (20 μm). Quantification of normalized current amplitudes from three independent experiments: (***A***) NMDA currents: ctrl = 1 ± 0.08, *n* = 30 neurons, hNR1 = 0.64 ± 0.06, *n* = 23 neurons, *p* = 0.0008; (***B***) GABA currents: ctrl = 1 ± 0.08, *n* = 31 neurons, hNR1 = 0.94 ± 0.09, *n* = 22 neurons; (***C***) kainate currents: ctrl = 1 ± 0.14, *n* = 21 neurons, hNR1 = 0.83 ± 0.15, *n* = 16 neurons. ***D***, ***E***, Synaptic responses were evoked by a brief somatic depolarization of neurons from −70 to 0 mV for 2 ms, synaptic NMDA currents were recorded in the presence of 0 mm Mg^2+^, 10 μm glycine, and 10 μm NBQX. Quantification of normalized current amplitudes from three independent experiments: (***D***) EPSCs: ctrl = 1 ± 0.13, *n* = 31 neurons, hNR1 = 0.99 ± 0.14, *n* = 23 neurons; (***E***) synaptic NMDA: ctrl = 1 ± 0.15, *n* = 26 neurons, hNR1 = 0.58 ± 0.12, *n* = 20 neurons, *p* = 0.039. Error bars indicate SEM. Unpaired *t* test was used to evaluate statistical significance. *p < 0.05, ***p < 0.001.

The lack of effect of hNR1 antibody on NMDA currents in cortical excitatory neurons was surprising, as previous studies have robustly shown that patients' CSF/IgG reduce NMDA currents of hippocampal pyramidal cells (Moscato et al., 2014; Kreye et al., 2016). To verify that the lack of effect on cortical neurons is not because of technical limitations of our autaptic assays, we repeated the autaptic recordings on hippocampal excitatory neurons. As reported previously (Kreye et al., 2016), treatment with hNR1 antibody selectively reduced whole-cell NMDA currents (36% decrease, ctrl = 1 ± 0.08, hNR1 = 0.64 ± 0.06, unpaired *t* test, *p* = 0.0008; Fig. 6*A*) and synaptic NMDA currents (43% decrease, ctrl = 1 ± 0.15, hNR1 = 0.58 ± 0.12, unpaired *t* test, *p* = 0.039; Fig. 6*E*) in hippocampal excitatory neurons, without affecting EPSC (Fig. 6*D*), GABA (Fig. 6*B*), and kainate (Fig. 6*C*) currents. Importantly, these results demonstrate that hNR1 antibody shows tissue-specificity, and in cortical cultures selectively affect inhibitory, and not excitatory, neurons.

### hNR1 antibody binds NMDARs within inhibitory synapses and reduce levels of inhibitory presynaptic proteins

**Figure 7.**
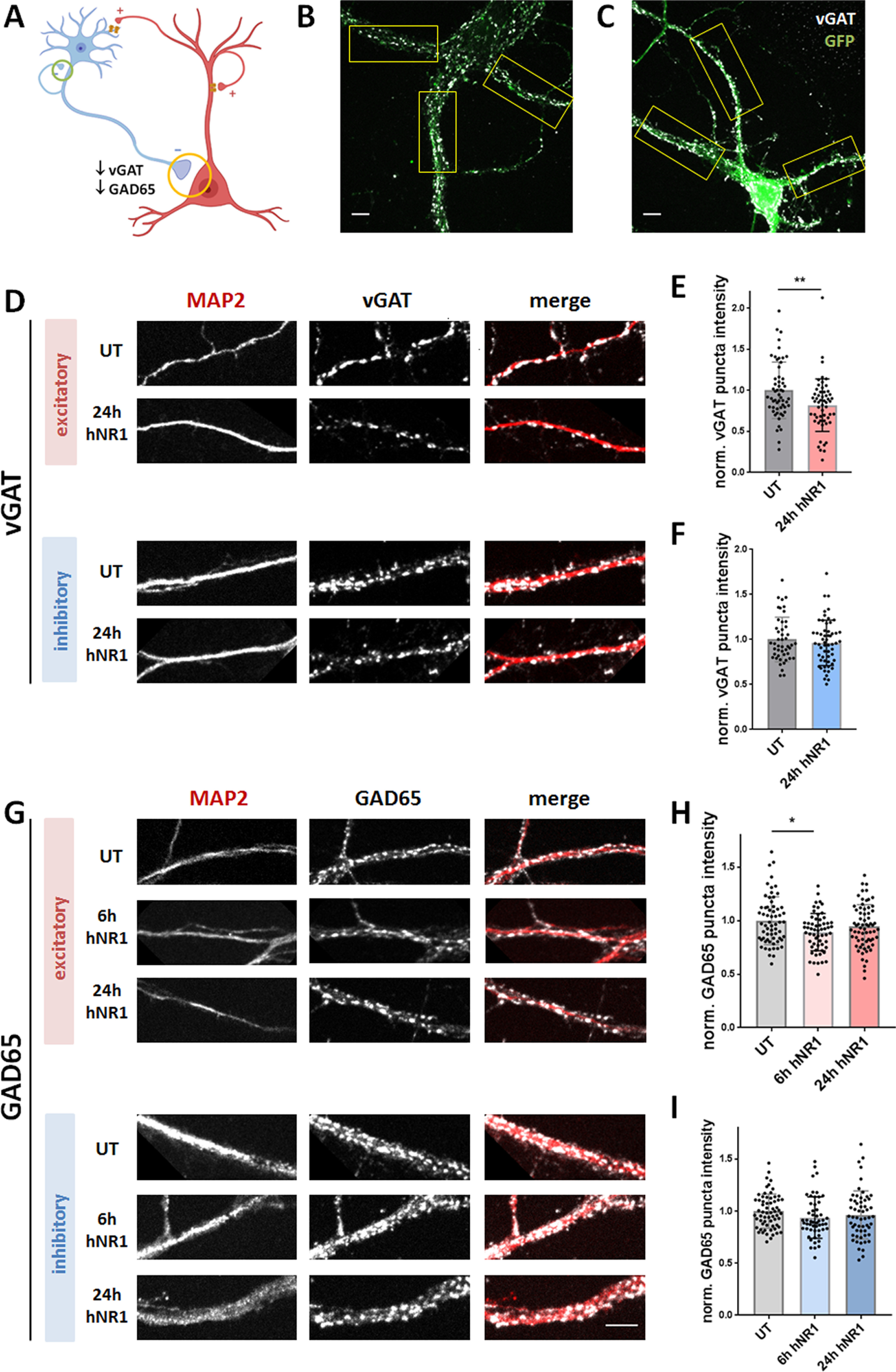
hNR1 antibody decreases levels of inhibitory presynaptic proteins VGAT and GAD65 in inhibitory-to-excitatory neuron synapses. ***A***, Schematic model showing investigated inhibitory synapses and summarizing the finding: decreased levels of GAD65 and VGAT at inhibitory synapses onto excitatory (yellow circle), but not inhibitory (green circle), neurons. ***B***, ***C***, Representative images of cortical excitatory (***B***) and inhibitory (***C***) neurons identified by staining with antibodies against GFP. Only proximal dendrites close to soma, which are easily identified as GFP-positive or GFP-negative, were chosen for ROI selection (boxes). ***D***, ***G***, Images of ROIs of cortical excitatory and inhibitory neurons which were fixed and stained with antibodies against GFP, MAP2, and either VGAT (***D***) or GAD65 (***G***), in untreated condition (UT) or after 6 or 24 h of hNR1 antibodies treatment. ***E***, ***F***, Normalized intensity of VGAT puncta along dendrites from three independent experiments: (***E***) on excitatory neurons: UT = 1 ± 0.05, *n* = 56 ROIs, 24-h hNR1 = 0.82 ± 0.04, *n* = 55 ROIs, *p* = 0.005; (***F***) on inhibitory neurons: UT = 1 ± 0.04, *n* = 47 ROIs, 24-h hNR1 = 0.96 ± 0.03, *n* = 57 ROIs. ***H***, ***I***, Normalized intensity of GAD65 puncta along dendrites from three independent experiments: (***H***) on excitatory neurons: UT = 1 ± 0.03, *n* = 64 ROIs, 6-h hNR1 = 0.89 ± 0.02, *n* = 59 ROIs, 24-h hNR1 = 0.95 ± 0.02, *n* = 67 ROIs, *p* = 0.01; (***I***) on inhibitory neurons: UT = 1 ± 0.02, *n* = 64 ROIs, 6-h hNR1 = 0.94 ± 0.03, *n* = 53 ROIs, 24-h hNR1 = 0.96 ± 0.03, *n* = 58 ROIs. Error bars indicate SEM. Scale bar: 10 µm. Paired *t* test (***E***, ***F***) and ANOVA Tukey's multiple comparison test (***H***, ***I***) were used to evaluate statistical significance. *p < 0.05, **p < 0.01.

The results from both neuronal networks (Fig. 4) and autaptic (Fig. 5) recordings suggest that hNR1 antibody causes a dysfunction of synaptic output of inhibitory neurons, represented by a reduced amplitude and/or frequency of IPSCs and mIPSCs. To explore possible presynaptic effects of the hNR1 antibody, we measured levels of proteins crucial for proper function of inhibitory presynapses before and after hNR1 antibody treatment by immunocytochemistry in cortical networks. Additionally, by employing GAD67-GFP mice, we distinguished between inhibitory synapses onto inhibitory (GFP-positive), and excitatory (GFP-negative) neurons (Fig. 7*B*,*C*). Two proteins are indispensable for proper neurotransmitter release within GABAergic presynapses: GAD65, which synthetizes GABA from glutamate within presynaptic boutons, and the vesicular GABA transporter (VGAT/VIAAT), which subsequently loads GABA into synaptic vesicles. Using quantitative immunocytochemistry, we measured the intensities of fluorescently labeled VGAT and GAD65 puncta along dendrites, to quantify their expression levels. Remarkably, a 24-h treatment with hNR1 antibody significantly reduced intensities of VGAT puncta (UT = 1 ± 0.05, 24-h hNR1 = 0.82 ± 0.04, unpaired *t* test, *p* = 0.005; Fig. 7*D*,*E*). Moreover, this decrease was specific to inhibitory synapses onto excitatory neurons, and not onto inhibitory neurons (Fig. 7*D*,*F*). On the other hand, the intensities of GAD65 puncta were unchanged after 24 h of hNR1 antibody treatment (Fig. 7*G–I*). We then additionally measured GAD65 expression levels after 6 h of hNR1 antibody treatment. At this time point, we observed a significant decrease in GAD65 puncta intensity (UT = 1 ± 0.03, 6-h hNR1 = 0.89 ± 0.02, 24-h hNR1 = 0.95 ± 0.02, ANOVA Tukey's multiple comparison test, *p* = 0.01; Fig. 7*H*), which again was specific to inhibitory synapses onto excitatory, but not inhibitory, neurons (Fig. 7*G–I*). Together, these data demonstrate that the hNR1 antibody reduces levels of the presynaptic proteins VGAT and GAD65, specifically in inhibitory-to-excitatory neuron synapses.

**Figure 8.**
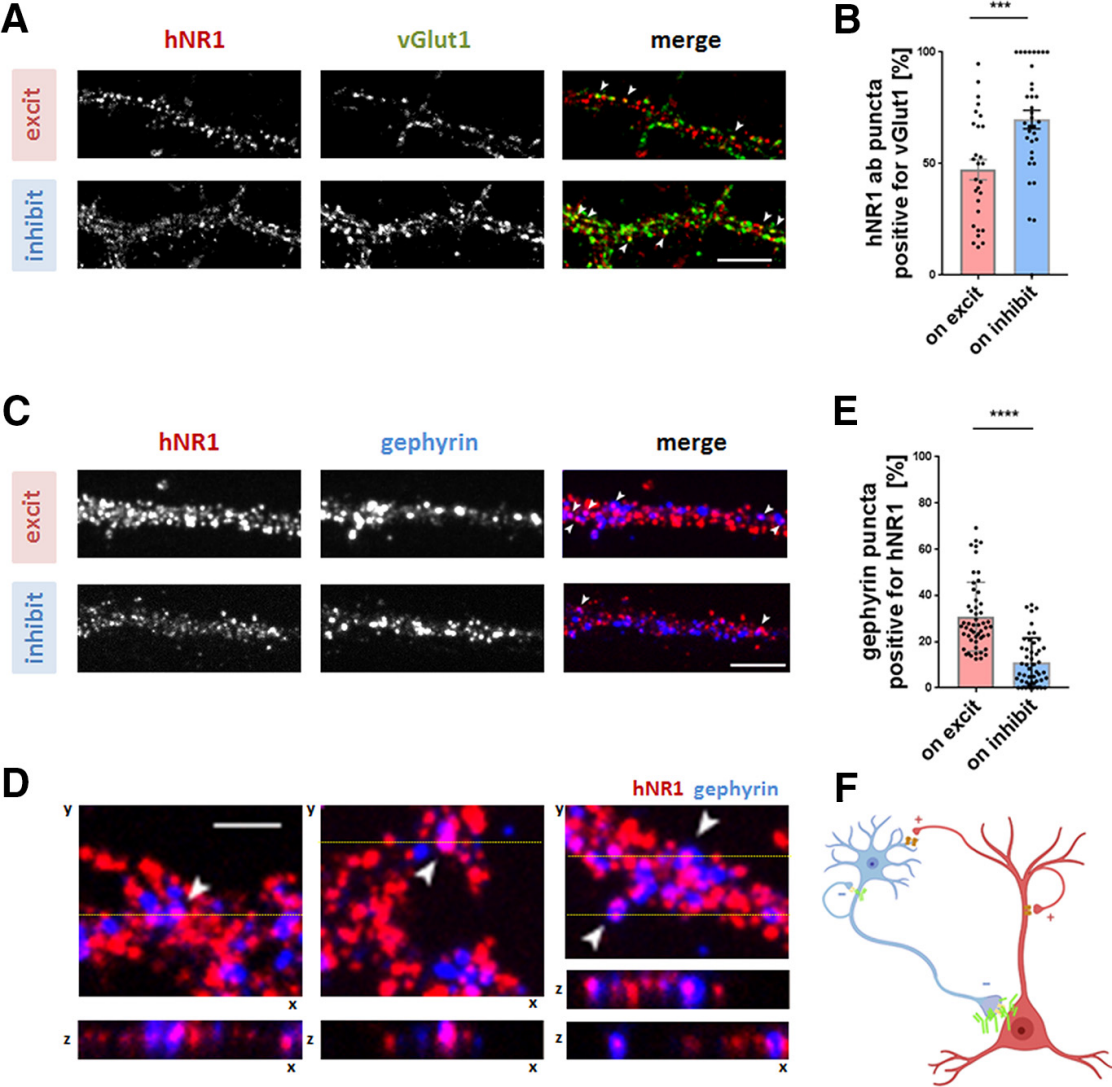
hNR1 antibody binds inhibitory synapses, preferentially on excitatory neurons. ***A***, Images of cortical neurons which were live stained with hNR1 antibody to detect acute binding pattern of the antibodies, and then fixed and co-stained for excitatory synaptic marker VGLUT1. Degree of colocalization of hNR1 antibody puncta and VGLUT1 puncta was used as a measure of excitatory synaptic or extrasynaptic staining. ***B***, Percentage of hNR1 antibody puncta positive for VGLUT1 (synaptic staining) per ROI: excitatory neurons = 47.28 ± 4.53, *n* = 28 ROIs, inhibitory neurons = 69.75 ± 4.19, *n* = 35 ROIs, 2 independent experiments, *p* = 0.006. ***C***, Images of cortical neurons live stained with hNR1 antibodies for acute hNR1 antibodies binding pattern, which were then fixed and co-stained with inhibitory synaptic marker gephyrin. Degree of colocalization between hNR1 antibody puncta and gephyrin puncta was used to assess inhibitory synaptic binding of the antibodies. Arrowheads indicate overlap between hNR1 and gephyrin. ***D***, Lower panel, 3D (z stack) volume images of individual inhibitory synapses from images in the upper panel at the level of yellow line, representing cross-section through 12 planes of z-stack. ***E***, Percentage of gephyrin puncta positive for hNR1 antibodies from three independent experiments: excitatory neurons = 30.84 ± 2.05, *n* = 53 ROIs, inhibitory neurons = 11.17 ± 1.52, *n* = 48 ROIs, *p* < 0.0001. ***F***, Schematic summarizing findings from these experiments: hNR1 antibody (green) binds within inhibitory synapses, preferentially onto excitatory neurons. Scale bar: 10 µm (***A***, ***C***) and 5 µm (***D***). Error bars indicate SEM. Unpaired *t* test was used to evaluate statistical significance. *p < 0.05, **p < 0.01, ****p < 0.0001.

**Figure 9.**
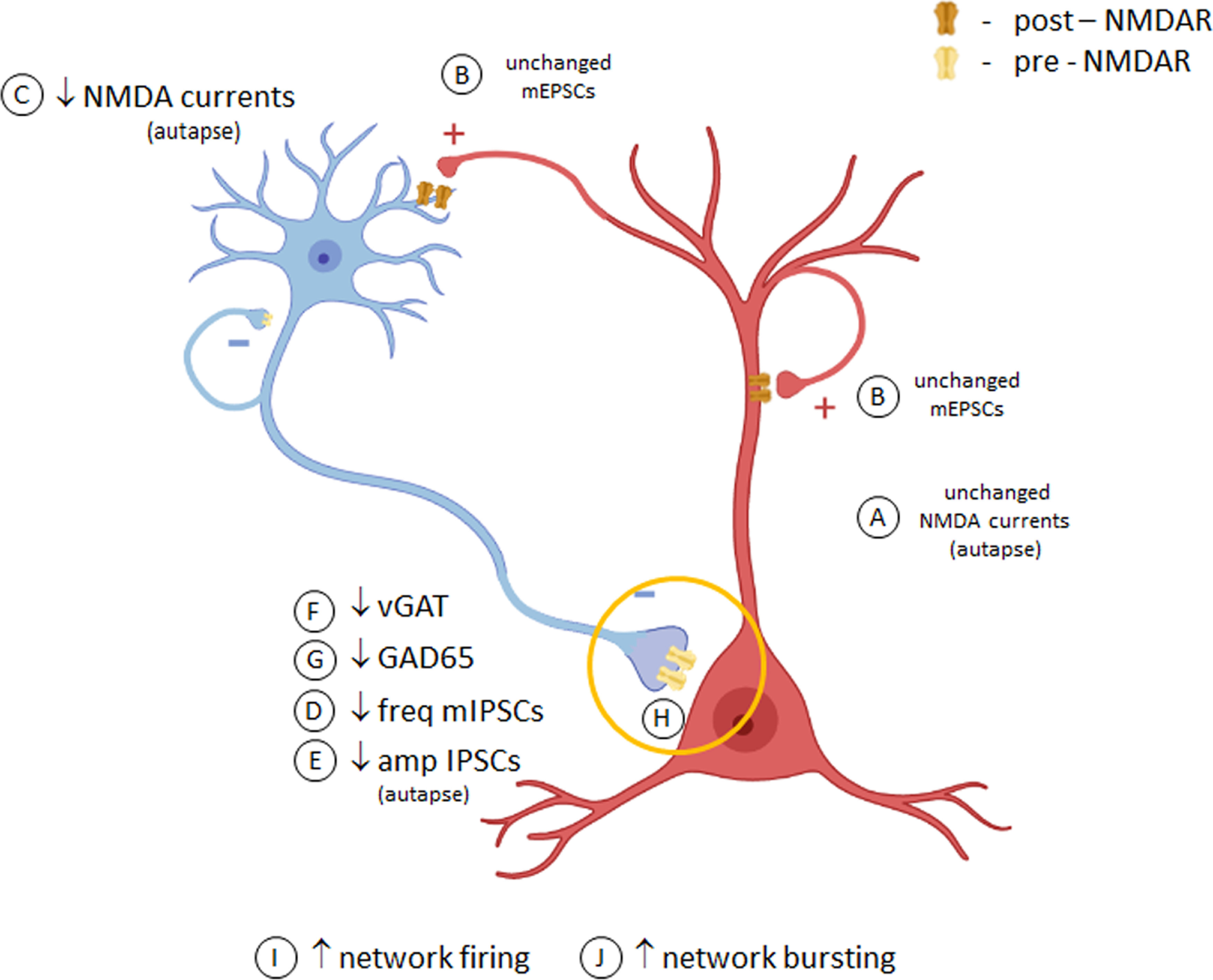
Speculative model of effects of hNR1 antibody on cortical inhibitory neuron and network function. In cortical neuronal cultures, hNR1 antibody does not affect NMDA currents (***A***) or synaptic transmission (***B***) of excitatory neurons, yet it selectively decreases NMDA currents on inhibitory cells (***C***). Antibody binding further impairs inhibitory synaptic transmission (***D***, ***E***) and decreases levels of GABA producing presynaptic proteins (***F***, ***G***), specifically in inhibitory-to-excitatory neuron synapses (yellow circle). Such reduced synaptic inhibitory output could result from altered transcriptional profiles of inhibitory neurons and/or local dysfunction of presynaptic NMDARs within these inhibitory synapses (***H***), mechanisms yet to be explored. This reduced inhibitory function disinhibits activity of excitatory neurons and the network as a whole (***I***, ***J***).

Finally, we asked whether the observed specific effect of hNR1 antibody on inhibitory neurons can be explained by a distinct binding pattern of the antibody in cortical cultures. One possibility could be that the antibody binds specifically to inhibitory neurons. Alternatively, the hNR1 antibody could target different subcellular pools of NMDARs, or even bind directly to inhibitory synapses to affect their function locally. We explored these options by analyzing the acute binding patterns of hNR1 antibody in our cortical cultures. First, live neurons were incubated with hNR1 antibody for 20 min at 10°C, washed, and incubated with anti-human secondary antibody for 20 min at 10°C. Cells were then fixed and co-stained for the excitatory presynaptic marker VGLUT1 to verify whether hNR1 antibody binds to excitatory synapses, as expected. The degree of co-localization of the hNR1 antibody puncta with VGLUT1 signal was used as a measure of excitatory synaptic versus extrasynaptic antibody binding. Although the hNR1 antibody decorated all neurons present in the culture in a punctate pattern, there was a striking difference between the binding patterns on excitatory and inhibitory neurons. While on inhibitory neurons most of hNR1 antibody puncta (70%) co-localized with VGLUT1, on excitatory neurons only half of the hNR1 antibody puncta co-localized with VGLUT1 (excitatory = 47.28 ± 4.53%, inhibitory = 69.75 ± 4.19%, unpaired *t* test, *p* = 0.006; Fig. 8*A*,*B*), suggesting that on excitatory neurons a larger pool of hNR1 antibody binds to structures outside of VGLUT1-positive synapses. Next, we asked whether hNR1 antibody can also bind NMDARs associated with inhibitory synapses. Here, neurons were co-stained with the hNR1 antibody and the inhibitory postsynaptic marker gephyrin to assess the degree of co-localization. Using confocal microscopy and z-stack analysis, we observed that hNR1 antibody indeed binds to a subpopulation of inhibitory synapses in our cortical cultures (Fig. 8*C*,*D*). Remarkably, up to 30% of inhibitory synapses on excitatory neurons bound the hNR1 antibody, whereas this phenomenon was much less common for inhibitory synapses on inhibitory neurons (excitatory = 30.84 ± 2.05%, inhibitory = 11.17 ± 1.52%, unpaired *t* test, *p* < 0.0001; Fig. 9*E*). This preference raises the possibility that the observed functional impact of the hNR1 antibody on inhibitory synapses may be because of the direct effects of this antibody on these synapses.

## Discussion

Our study demonstrates that a patient-derived antibody targeting the NR1 subunit of NMDARs adversely affects the function of cortical neuronal networks, driving them into a hyperactive state (Figs. 1, 3). This increased excitability seems to be a result of reduced inhibitory neuron output onto excitatory neurons, as indicated by reduced amplitudes of IPSCs, lowered frequency of network mIPSCs and decreased levels of inhibitory presynaptic proteins (Fig. 9). This contrasts with the effects of this antibody on hippocampal neurons, where it primarily affects NMDAR function on excitatory neurons (Fig. 6; Kreye et al., 2016). Moreover, in cortical networks hNR1 antibody interferes with NMDAR-Npas4-mediated mechanisms of stabilizing network excitability (Fig. 2). These observations provide insights into how such autoantibodies can differentially affect hippocampal versus cortical circuits, giving rise to impaired memory function or psychiatric symptoms in patients with NMDAR encephalitis, respectively.

### hNR1 antibody increases cortical network activity

Our primary observation is that hNR1 antibody causes a hyperexcitable state of cortical networks (Figs. 1, 3). Although counterintuitive, since NMDAR hypofunction should dampen glutamatergic transmission, our data are in line with clinical and molecular studies showing that seizures occur in ∼60% of patients with NMDAR encephalitis (de Bruijn et al., 2019) and that seizure susceptibility is increased in mice exposed to patient CSF (Wright et al., 2015).

Intriguingly, in contrast to our findings (Fig. 1), two groups used MEAs to assess effects of patient CSF on cortical (Jantzen et al., 2013) or hippocampal (Koch et al., 2019) neuronal networks and reported a relative decrease in activity. Such differences could arise from varied approaches used. Both studies examined the acute effects of antibodies (10–15 min), using patient CSF containing a mixture of antibodies. In contrast, our 46-h recording revealed that the presence of the hNR1 antibody leads to a gradually increasing activity, however, over 24 h and not 15 min. Mechanistically, this occurs in a timeframe corresponding to the rate of antibody-induced receptor internalization both described previously (Moscato et al., 2014) and observed for hNR1 (data not shown). Thus, the increase in cortical network activity by the hNR1 antibody seems to be a relevant causal mechanism for both hyperexcitability and epilepsy in patients.

A fundamental question raised by our MEA analysis is how the hNR1 antibody leads to a steady increase in network spiking? One possibility is an adaptive response of the network to a partial block of NMDARs. Blocking half of surface NMDARs (1 μm AP5) lead to a modest but nonsignificant increase in network spiking, indicating that it is possible, yet unlikely, that a partial block of surface NMDARs contributes to the seen increase in network activity. Rather, these data suggest that hNR1 antibody might instead act through specific receptors/neuronal subtypes to disturb E/I balance. Indeed, in the presence of hNR1 antibody, cortical networks dramatically increase their bursting activity (Fig. 1*J–P*) and loose responsiveness to bicuculline (Fig. 3*A–F*), suggesting that the inhibitory system is likely affected.

A second puzzling question is why neuronal spiking continues to climb in the presence of hNR1 antibody, when one would expect mechanisms stabilizing network excitability, such as Npas4 signaling (Bloodgood et al., 2013), to counter a hyperactive state. Surprisingly, nuclear Npas4 levels did not increase in the presence of hNR1 antibody (Fig. 2*A–D*), suggesting that this mechanism became ineffective. Accordingly, normal NMDA-mediated increase in nuclear Npas4 was blocked by addition of hNR1 antibody (Fig. 2*E*,*F*). This could be because of a direct effect of hNR1 antibody on NMDARs, as AP5 also prevented the induction of Npas4 after NMDA application (Fig. 2*E*,*F*). Together, these data indicate that the hNR1 antibody can interfere with homeostatic mechanisms regulated by NMDAR/Npas4 signaling.

### hNR1 antibody impairs the output of cortical inhibitory neurons

While the depression of NMDAR/Npas4 signaling may explain why hyperexcitability is not scaled down, it does not fully explain why the activity increases in the first place. One possibility is that hNR1 antibody acts on a subset of receptors, synapses or neurons. In the cortex, a correct E/I balance is maintained by GABAergic interneurons orchestrating excitability of pyramidal cells. Our data indicate that cortical GABAergic interneurons are indeed a primary target of hNR1 antibody, specifically altering the output of inhibitory synapses onto excitatory neurons. Consistently, hNR1 antibody treatment decreased action potential-independent, tonic inhibitory drive onto excitatory neurons, measured by frequency of mIPSCs (Fig. 4*A–F*). This was specific to inhibitory synapses, as mEPSCs frequency remained unchanged (Fig. 4*I–P*). This is further supported by a decrease in the levels of both VGAT and GAD65 in presynaptic inhibitory boutons formed onto excitatory neurons, but not inhibitory interneurons (Fig. 7).

Importantly, cell-autonomous autaptic recordings confirmed the specific impact of hNR1 antibody on inhibitory neuron function, where it significantly reduced NMDA– and evoked synaptic currents exclusively in inhibitory autaptic neurons (Fig. 5). Although reduced NMDA currents are likely because of receptor internalization, how loss of surface NMDARs translates into reduced inhibitory synaptic transmission remains elusive. Conceivably, reduced NMDAR-mediated Ca^2+^ influx could trigger changes in transcriptional profiles in these neurons, leading to decreased levels of presynaptic proteins (Fig. 7) and ultimately synaptic output (Fig. 5), similarly to networks in which excitation has been removed (Lau and Murthy, 2012). However, given that we observed increased somatic calcium signals in all cortical neurons (Fig. 3), whether such mechanisms are involved remains unclear and further RNA sequencing studies are needed to elucidate possible cell type-specific transcriptional changes.

### hNR1 antibody shows brain regional specificity

Remarkably, our data imply that autoantibodies can have different effects in varying brain regions. In cortex, we find that hNR1 specifically impairs cortical inhibitory, but not excitatory, neurons. This contrasts with studies in the hippocampus, showing that these autoantibodies primarily affect excitatory neuron function (Fig. 6; Hughes et al., 2010; Kreye et al., 2016). This regional specificity indicates that there are limitations with current preclinical models, which passively deliver patient CSF/antibodies into ventricles (Planagumà et al., 2016; Taraschenko et al., 2019) or hippocampus (Würdemann et al., 2016; Kersten et al., 2019). While this approach can provide direct antibody access to hippocampal structures, they rarely reach cortical regions (Planagumà et al., 2015). As such, active immunization models which more often report increased sensitivity to seizures (Jones et al., 2019; Wagnon et al., 2020) may provide for a more clinically-relevant distribution of antibodies and thus a better understanding of their region-specific effects.

### hNR1 antibody binds specific pools of NMDARs and affects inhibitory synapses

At present, it is unclear which mechanisms lead to brain regional and/or neuronal type specificity of hNR1 antibody. One possibility could be that the hNR1 antibody binds variably on excitatory versus inhibitory neurons. Acute antibody binding studies supports this idea, as hNR1 antibody differentially binds synaptic and extrasynaptic NMDARs on inhibitory versus excitatory cortical neurons (Fig. 8*A*,*B*). This is relevant as synaptic and extrasynaptic NMDARs trigger distinct downstream signaling pathways and could therefore contribute to differential effects on each cell type.

An intriguing complementary mechanism could involve local effects of hNR1 antibody on presynaptic NMDARs. Although controversial and debated in the field, presynaptic NMDARs within inhibitory synapses have been shown to control neurotransmitter release (Bouvier et al., 2015) and regulate inhibitory drive onto pyramidal neuron in prefrontal cortex (Mathew and Hablitz, 2011; Pafundo et al., 2018). Thus, their hypofunction because of hNR1 binding could partially explain the observed decrease in mIPSCs (Fig. 4). Accordingly, we observed that the hNR1 antibody indeed decorates inhibitory synapses (Fig. 8). While it is unclear whether this binding is presynaptic or postsynaptic, a recent immuno-EM study found that the hNR1 antibody could indeed decorate presynaptic-NMDARs (Wagner et al., 2020). Importantly, studies by Fiszman et al. (2005) reported that reducing presynaptic-NMDAR signaling decreases the size of presynaptic inhibitory boutons, as measured by GAD65 puncta intensity, similar to our observations (Fig. 7). Thus, in addition to more general effects of the hNR1 antibodies on inhibitory interneurons, there is an emerging framework for how these antibodies could also have more selective actions on subsets of inhibitory synapses, which ultimately alter the excitability of cortical networks in patients with NMDAR encephalitis.

### Relevance for neuropsychiatric disorders

Remarkably, NMDAR hypofunction on cortical interneurons has long been implicated to underlie cortical disinhibition and core psychiatric symptoms in schizophrenia (Nakazawa et al., 2012). Genetic deletion of NMDARs in interneurons *in vivo* produces schizophrenia-like behaviors (Belforte et al., 2010), impairs E/I balance and increases excitability of cortical pyramidal cells (Pafundo et al., 2021). Similarly, ketamine impairs mIPSCs, without affecting mEPSCs, and leads to increased pyramidal excitability in prefrontal cortical slices (Zhang et al., 2008), which is strikingly similar to our effects after hNR1 antibody treatment (Figs. 1, 3, 4). Our data thus provides evidence that patient-derived autoantibodies can disturb similar pathways and suggest a possible common mechanism across neuropsychiatric disorders.

Importantly, the proposed mechanisms of antibody action are not mutually exclusive and may act in a complementary manner to exert regional changes in synaptic plasticity, subunit-specific transcriptional changes and presynaptic regulation of inhibitory drive to produce complex behavioral and cognitive phenotypes. Together, our data reveal a novel, region-specific mechanism of anti-NMDAR antibodies in cortical neurons causing inhibitory dysfunction and network disinhibition.
